# Real‐Time Wireless Detection of Heavy Metal Ions Using a Self‐Powered Triboelectric Nanosensor Integrated with an Autonomous Thermoelectric Generator‐Powered Robotic System

**DOI:** 10.1002/advs.202410424

**Published:** 2024-11-08

**Authors:** Yan‐Tsz Huang, Arshad Khan, Anindita Ganguly, Kuldeep Kaswan, Sreerag Suresh, Yu‐Ying Cheng, Kuan‐Ming Lee, Jui‐Han Yu, Zong‐Hong Lin

**Affiliations:** ^1^ Department of Biomedical Engineering National Taiwan University Taipei 10167 Taiwan; ^2^ Institute of Biomedical Engineering National Tsing Hua University Hsinchu 30013 Taiwan; ^3^ International Intercollegiate PhD Program National Tsing Hua University Hsinchu 30013 Taiwan; ^4^ Institute of NanoEngineering and MicroSystems National Tsing Hua University Hsinchu 30013 Taiwan; ^5^ Department of Power Mechanical Engineering National Tsing Hua University Hsinchu 30013 Taiwan

**Keywords:** heavy‐metal ions, intelligent monitoring, robotic hand, self‐powered, thermoelectric generator, triboelectric nanosensor

## Abstract

The integration of the Internet of Things (IoT) with advanced sensing technologies is transforming environmental monitoring and public health protection. In this study, a fully self‐powered and automated chemical sensing system is developed and integrated with a robotic hand for “touch and sense” detection of toxic heavy metal ions (Pb^2^⁺, Cr⁶⁺, As^3^⁺) in aquatic environments. The system combines a self‐powered solid‐liquid triboelectric nanosensor (SL‐TENS) with a thermoelectric generator (TEG), which harnesses ambient heat to power the robotic hand, eliminating the need for external power sources. The robotic hand is controlled wirelessly via an exo‐hand, minimizing the risk of exposure during remote monitoring. The sensing component uses copper oxide nanowires (CuO NWs) coated with ion‐selective membranes (ISMs) to enhance triboelectric output and enable highly selective ion detection. The system demonstrates effective real‐time, on‐site detection in lake water and data transmitted wirelessly to the user. This innovative approach provides a highly safe and efficient method for detecting hazardous pollutants in difficult‐to‐access areas, offering significant potential for wireless and real‐time environmental monitoring and hazard prevention, thus contributing to the safeguarding of human health. This study presents a novel advancement in the field of IoT‐enabled environmental monitoring systems.

## Graphical abstract

This study introduces a self‐powered environmental monitoring system that integrates a triboelectric nanosensor (TENS) with a robotic hand, controlled remotely by an exo‐hand and powered by a thermoelectric generator (TEG). The fully autonomous system detects Pb^2^⁺, Cr⁶⁺, and As^3^⁺ ions in real‐time and operates without external power, ideal for remote water quality monitoring in hazardous environments.

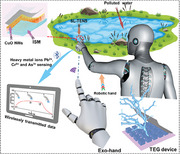

## 1 Introduction

The integration of the Internet of Things (IoTs) into modern life has revolutionized our interaction with the world around us.^[^ ^1^, ^2^ ^]^ By seamlessly connecting devices and systems across a myriad of applications ranging from healthcare and environmental monitoring to smart agriculture, home security, and wearable electronics, IoTs enable real‐time data collection and communication, enhancing efficiency and convenience.^[^ ^3^, ^4^, ^5^, ^6^ ^]^ At the core of this transformation are sensors, which serve as the critical components that gather and transmit the data necessary for IoT networks to function effectively. A variety of sensors, including optical, pressure, chemical sensors, temperature, and motion sensors have been developed to target specific applications. The integration of IoT with chemical sensors, in particular, allows for the early detection of pollutants and hazardous chemicals, enabling prompt interventions to safeguard public health.^[^ ^7^, ^8^, ^9^ ^]^ Among the pollutants generated by human activities, heavy metal ions such as lead (Pb^2^⁺), chromium (Cr⁶⁺), and arsenic (As^3^⁺) pose serious threats even at low concentrations due to their high toxicity, prolonged environmental persistence, high mobility in water, and propensity for bioaccumulation in living organisms.^[^ ^10^, ^11^, ^12^ ^]^ The detection of these ions has been demonstrated by wearable chemical sensing systems.^[^ ^13^ ^]^ However, detecting these heavy metal ions using wearable chemical sensors often exposes the human operator to the risk of toxicity.

Further advancements in IoT technology have driven the development of sensing systems that incorporate chemical sensors with robotic technology for remote sensing, effectively minimizing the risk of exposure to toxic chemicals. In recent years, studies have demonstrated that robot‐assisted enzyme‐based electrochemical sensors can detect a wide range of substances, including food flavors, organophosphates, 2,4,6‐trinitrotoluene (TNT), and the SARS‐CoV‐2 virus.^[^ ^14^, ^15^, ^16^ ^]^ However, these chemical sensors typically rely on external power sources for their operation, necessitating the need to develop self‐powered sensors to address these challenges.^[^ ^17^, ^18^, ^19^ ^]^ Energy harvesting technologies, such as nanogenerators, have emerged as promising solutions for self‐powering options by harnessing energy from the surrounding environment. One notable example is triboelectric nanogenerators (TENGs), which have gained significant attention as eco‐friendly energy harvesting devices.^[^ ^20^ ^]^ TENGs convert mechanical energy into electrical energy through the phenomena of contact electrification and electrostatic induction. This process happens when two materials come into contact and then separate, generating charges on their surfaces.^[^ ^21^, ^22^ ^]^ The electrical energy produced can then power various systems, eliminating the need for external power sources.^[^ ^23^, ^24^ ^]^ When the surface of a TENG is modified with specific materials or receptors to detect various physical and chemical parameters, it is termed a triboelectric nanosensor (TENS).^[^ ^25^, ^26^ ^]^ These sensors leverage the same principles as TENGs but are tailored for sensing applications. The interaction between the TENG's surface and the target analytes alters the surface charge, which is then used to detect changes in the system. Research has predominantly focused on solid‐solid TENS for detecting a wide range of analytes and contaminants.^[^ ^27^, ^28^, ^29^, ^30^, ^31^, ^32^ ^]^

However, fabrication of these solid‐solid TENS is challenging, including maintaining durability, sensitivity, and long‐term stability. To address these issues, researchers and scientists are investigating the use of liquids as contact materials in TENS. Liquid layers offer advantages such as availability, cost‐effectiveness, and sustainability.^[^ ^33^, ^34^, ^35^, ^36^ ^]^ Additionally, they act as lubricants, promoting smooth interactions and enhancing sensor reliability. The liquid layer absorbs varying concentrations of analytes, causing changes in the surface charge and enabling self‐powered chemical sensing through contact separation. Solid‐liquid TENS (SL‐TENS) thus represents a promising approach for creating robust and stable self‐powered nanosensors without relying on external power sources.^[^ ^37^ ^]^ For instance, self‐powered SL‐TENS have been successfully used to detect analytes such as dopamine, E. coli, mercury ions, and catechin in green tea.^[^ ^26^, ^33^, ^34^, ^36^ ^]^ The use of thermoelectric generators (TEGs) to power various systems has gained significant attention.^[^ ^38^ ^]^ TEGs are capable of harnessing minor temperature differences in the environment such as those found in indoor buildings, running vehicles, electronic devices, or even the human body to convert heat energy into electrical energy.^[^ ^39^, ^40^, ^41^, ^42^ ^]^ By harvesting abundant heat sources, TEGs can provide a continuous power supply to robotic hands, effectively eliminating the need for regular battery recharges or replacements. This approach not only enhances the efficiency and operational time of robotic systems but also fulfills the growing demand for fully self‐powered and automated devices that leverage ambient energy. The integration of TEGs into robotic hands represents a significant step toward developing autonomous and sustainable systems that can function independently of external power sources, making them ideal for long‐term applications in various environments.

In this study, we have developed a fully self‐powered and automated chemical sensing system, wherein Pb^2+^, Cr^6+^, and As^3+^ ISM‐based self‐powered SL‐TENS were integrated with the fingers of a robotic hand for “touch and sense” fingertip detection of toxic Pb^2+^, Cr^6+^, As^3+^ ions. Next, a TEG module has been introduced, which harvests energy from the environment to power the robotic hand thus eliminating the need for any external power supply. Most notably, the robotic hand was connected to an exo‐hand (a glove‐like exoskeleton) wirelessly for remote monitoring. The exo‐hand remotely controls the actions of the robotic hand through the wi‐fi function, which prevents the user from even being close to the robotic hand while detecting the toxic heavy metal ions. The sensing component is designed using copper oxide nanowires (CuO NWs) grown on copper substrate and coated with different ion‐selective membranes (ISMs) for the detection of respective ions. The CuO NWs offer several advantages over other semiconductor materials due to their excellent triboelectric properties, cost‐effectiveness, and abundance.^[^ ^32^, ^43^, ^44^ ^]^ Additionally, their chemical stability in aqueous environments and easy synthesis process makes them ideal for efficient sensing applications.^[^ ^32^, ^45^, ^46^ ^]^ The as‐fabricated sensors served as the solid contact material layer to enhance the triboelectric output due to the highly selective binding affinity of the electrodes with the ions. The DI water was used as a contact liquid. To comprehensively examine the fundamental principles underlying solid‐liquid interface sensing, the effects of surface potential and work function on contact electrification were also demonstrated.

Furthermore, the wireless remote and real‐time detection capability of the automated chemical sensing system in real environment lake water was also investigated. The real sample was directly utilized as contact liquid for the detection of Pb^2+^, Cr^6+^ As^3+^ ions. The generated data was recorded and transmitted wirelessly to the user's device through a Bluetooth transmission system. To the best of our knowledge, the SL‐TENS integrated with robotic system which is powered by TEG for highly specific, sensitive, and safe detection of Pb^2+^, Cr^6+^, and As^3+^ ions has not been reported so far. Overall, this innovative and intelligent monitoring system is ideal for detecting pollutants in difficult‐to‐access areas, real‐time monitoring of aquatic environments, and hazard monitoring, thereby safeguarding human health.

## 2 Results and Discussion

### 2.1 Pb^2+^, Cr^6+^, and As^3+^ ISMs‐based SL‐TENS Fabrication and Their Integration with Robotic Hand

**Figure 1:**
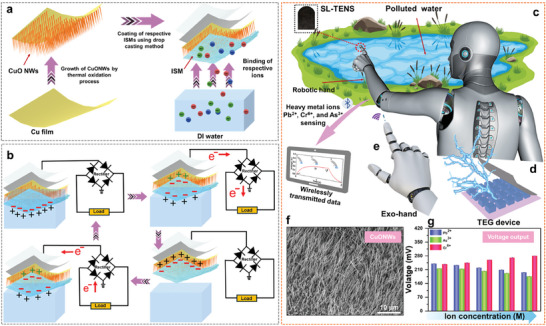
Solid–Liquid TENS incorporated with robot hand for the detection of Pb^2+^, Cr^6+^, and As^3+^ heavy metal ions. a) Schematic showing the fabrication process of Pb^2+^, Cr^6+^, and As^3+^ ISMs‐based robotic hand SL‐TENS from highly flexible and ultrathin copper foil. b) Illustration of working mechanism of Pb^2+^, Cr^6+^, and As^3+^ ISM based SL‐TENS. c) Schematic illustration of the fingertip‐shaped SL‐TENS contacting and separating with the real sample for detection of Pb^2+^, Cr^6+^, and As^3+^ ions. d) A thermoelectric generator device for powering the robot hand by harvesting environmental heat energy. e) Wireless control of the robotic hand by the exo‐hand. f) The FESEM image of CuO NWs grown on the Cu film shows a uniform distribution of hair‐like structure. g) Voltage output data obtained from the sensing of the Pb^2+^, Cr^6+^, and As^3+^ ions in polluted water by the respective robotic hand‐based sensors.

Pb^2+^, Cr^6+^ and As^3+^ ISMs‐based SL‐TENS were fabricated in the shape of the tip of the robot hand's finger for self‐powered and automated detection of heavy metal ions like Pb^2+^, Cr^6+^, and As^3+^. **Figure** **1**a demonstrates the fabrication process of the robotic hand‐based SL‐TENS from highly flexible and ultrathin copper foil. A dense array of CuO NWs was grown on the surface of the copper foil by a thermal oxidation process in which copper foil is heated at a high temperature of 500 °C for 5 h. Following the growth of CuONWs, a layer of respective ISMs (Pb^2+^, Cr^6+^, and As^3+^ ISMs) was drop cast for highly selective detection of Pb^2+^, Cr^6+^, and As^3+^ ions. The next step shows the overview of the sensing process for the detection of the Pb^2+^, Cr^6+^, and As^3+^ ions by the Pb^2+^, Cr^6+^, and As^3+^ ISMs‐based robotic hand SL‐TENS. The working principle of different Pb^2+^, Cr^6+^, and As^3+^ ISM‐based SL‐TENS, as illustrated in Figure 1b, relies on contact electrification and electrostatic induction. When the surface of ISMs comes into contact with the liquid DI water, electrons transfer from DI water to the surface of ISMs, making the DI water positively charged and the surface of ISMs negatively charged. As the ISMs surface is withdrawn, a potential difference arises, causing electrons to flow through an external circuit to maintain electrical neutrality. When the surfaces recontact, the flow reverses. These periodic contact‐separation movements generate alternating currents, which results in continuous varying output voltage. For the detection of the Pb^2+^, Cr^6+^, and As^3+^ heavy metal ions in the polluted water using the solid–liquid contact electrification mechanism, the as‐fabricated sensors were integrated with the tips of fingers of the robot hand as illustrated in Figure 1c. The ISM coated on the sensor functions as the sensing probe and triboelectric layer, which can generate an electrical output spontaneously following successive contact and separation with the contact liquid. The inset image in Figure 1c shows the real image of the ISM‐modified CuONWs grown on the Cu film SL‐TENS in the shape of the fingertip of the robot hand. The CuO NWs were also grown on a 10 × 10 cm Cu foil which demonstrates the large‐scale production potential of the different SL‐TENS, as shown in Figure S1 (Supporting Information). The SL‐TENS operated under a single electrode configuration where copper (Cu) substrate serves as the electrode. The obtained data was transmitted wirelessly to a laptop or tablet device of the user by a printed circuit board (PCB) equipped with a built‐in Bluetooth low energy (BLE) function. As demonstrated in Figure 1d, a TEG device was utilized to power the robotic hand by harvesting environmental heat energy to realize a fully self‐powered chemical sensing system, avoiding the use of any external power source. Figure 1e shows the wireless control of the robotic hand by the exo‐hand for remote monitoring. The Field Emission Scanning Electron Microscopy (FESEM) of the solid–liquid TENS reveals the morphology of the CuO NWs grown on the Cu film, as demonstrated by the uniformly distributed hair‐like structure in Figure 1f. Figure 1g demonstrates the triboelectric output data obtained from the contact separation of the robotic hand‐based sensor with the polluted water sample to detect respective Pb^2+^, Cr^6+^, and As^3+^ heavy metal ions. The triboelectric output is utilized as the signal for the detection, and its magnitude depends upon the concentration of the Pb^2+^, Cr^6+^, and As^3+^ heavy metal ions on the surface of Pb^2+^, Cr^6+^, and As^3+^ ISMs, respectively.

### 2.2 Material Characterization of the ISMs Triboelectric Layer Reacted with the Heavy Metal Ions

**Figure 2:**
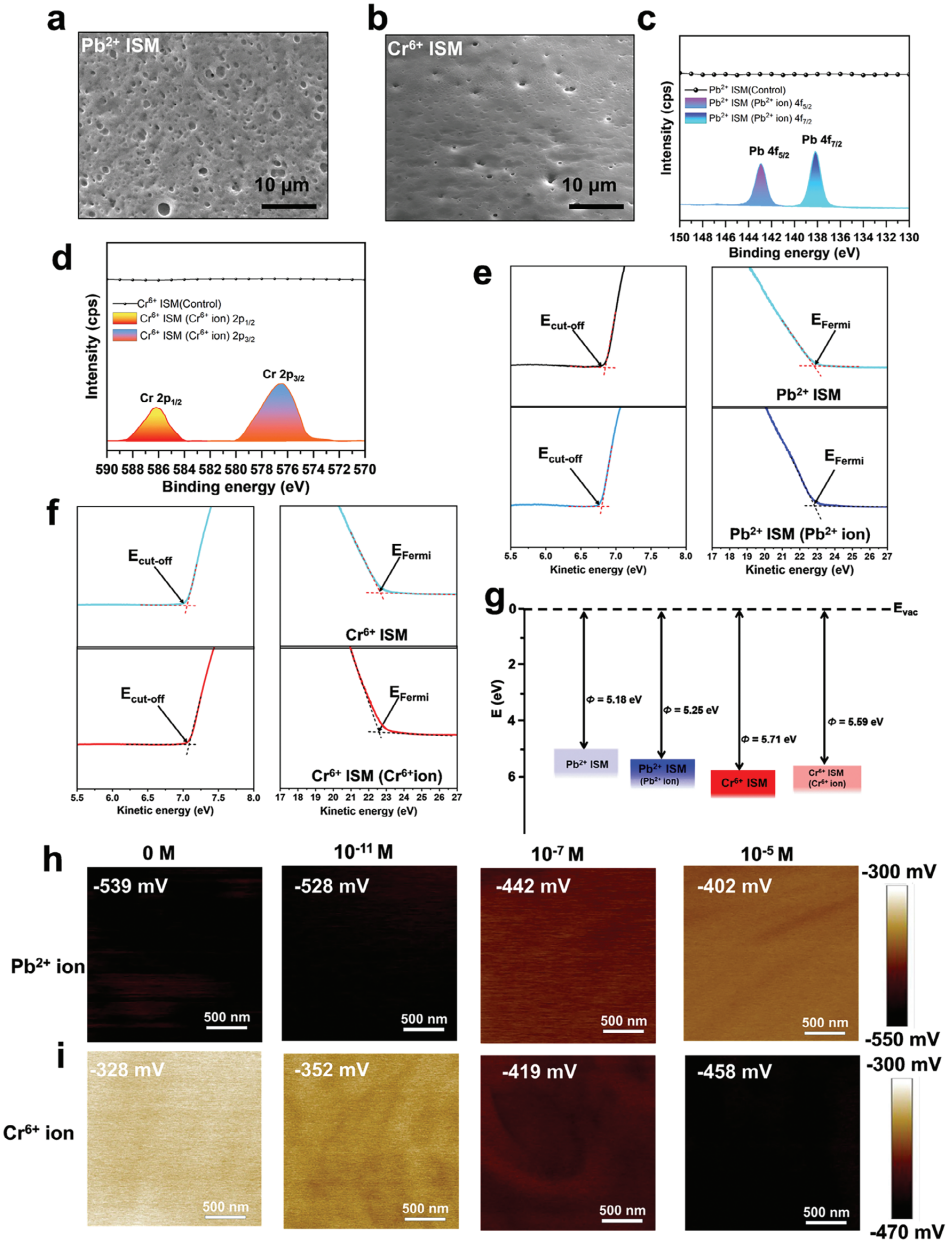
Characterization of the Pb^2+^, Cr^6+^, and As^3+^ ISMs before and after reaction with their respective heavy metal ions. a,b) Surface morphology of the Pb^2+^ and Cr^6+^ ISMs showing a uniform film with porous structures revealed by FESEM. c,d) High‐resolution XPS showing Pb 4f peaks and Cr 2p peaks at (138, 143 eV) and (578.3, 587.8 eV) binding energy positions after reaction of Pb^2+^and Cr^6+^ ISMs with 10^−5^ m concentration of respective ions. e,f) The UPS spectra of Pb^2+^ and Cr^6+^ ISMs and after their binding with Pb^2+^ and Cr^6+^ ions, respectively. The positions of the indicated Fermi energy (*E*_Fermi_) and the secondary electron cutoff (*E*_Cut‐off_) are utilized to calculate the work functions (Φ) of the materials. g) A graphical illustration showing the alteration in the work function of Pb^2+^ and Cr^6+^ ISMs after binding with Pb^2+^ and Cr^6+^ ions. The work function reduction signifies that less energy is needed for electrons to cross the barrier of surface potential and vice versa. h,i) Surface potentials of Pb^2+^ and Cr^6+^ ISMs measured by KPFM after their binding with varying concentrations of Pb^2+^ and Cr^6+^ ions (0, 10^−11^, 10^−7^, 10^−5^ m).

To demonstrate the effective reaction of the Pb^2+^, Cr^6+^, and As^3+^ heavy metal ions to respective ISM layers such as Pb^2+^, Cr^6+^, and As^3+^ ISMs, we systemically performed the structural, morphological, and compositional characterization. The FESEM characterization of the surface topography of the fabricated sensors revealed the surface morphology of ISMs. As demonstrated in **Figures** **2**a,b, Pb^2+^ and Cr^6+^ ISMs form a uniform film with porous structures. The surface morphology of As^3+^ ISM with a uniform film of porous structure is shown in Figure S2a (Supporting Information). A high‐resolution X‐ray photoelectron spectroscopy (XPS) analysis was performed to verify the efficient binding of heavy metal ions on the surface of their corresponding ISMs. The XPS spectra show the appearance of Pb 4f peaks at 138 and 143 eV binding energy positions representing Pb 4f_7/2_ and Pb 4f_5/2_ spin‐orbit spin components, respectively (Figure 2c).^[^ ^47^ ^]^ As depicted in Figure 2d, the effective binding of the Cr^6+^ ion with Cr^6+^ ISM gives peaks at 578.3 and 587.8 eV binding energy positions representing Cr 2p_3/2_ and Pb 2p_1/2_ spin‐orbit spin components, respectively.^[^ ^48^ ^]^ The XPS spectra also showed As 3d_3/2_ and As 3d_5/2_ peaks at 45.8 and 44.7 eV binding energy positions, respectively as depicted in Figure S2b (Supporting Information).^[^ ^49^ ^]^ Ultraviolet photoelectron spectroscopy (UPS) characterization of the Pb^2+^, Cr^6+^, and As^3+^ ISMs after reacting with 10^−5^ m concentration of their respective metal ions was performed to measure the change in the work function (Figures 2e,f; Figure S3a, Supporting Information). The calculated work functions of Pb^2+^, Cr^6+^, and As^3+^ ISMs were 5.18, 5.71, and 5.28 eV. However, after reaction with Pb^2+^, Cr^6+^, and As^3+^ respectively, the work functions of Pb^2+^ and As^3+^ ISMs were increased to 5.25 and 5.91 eV, and the work function of the Cr^6+^ ISM was decreased to 5.59 eV (Figure 2g; Figure S3b, Supporting Information). Further, the surface potential of the sensing surface is a critical factor that can affect the output performance of a triboelectric nanosensor.^[^ ^50^, ^51^ ^]^ Therefore, Kelvin probe force microscopy (KPFM) was employed to capture the surface potential images and analyze their corresponding Gaussian distributions for the unmodified Pb^2+^, Cr^6+^, and As^3+^ ISMs, as well as after their exposure to Pb^2^⁺, Cr⁶⁺, and As^3^⁺ ions, respectively. The surface potential analysis of Pb^2+^ and As^3+^ ISMs showed similar trends, that is, the values increased gradually after being reacted with an increasing concentration of their respective ions. However, the surface potential of Cr^6+^ ISM showed a decreasing trend upon reaction with Cr^6+^ ions in increasing concentration. These results are in agreement with the calculated work functions. Initially, the surface potential of the Pb^2+^ ISM was recorded to be −539 mV, which increased to −402 mV after reacting with 10^−5^ m of Pb^2+^ ions (Figure 2h). The corresponding Gaussian distribution is shown in Figure S4a (Supporting Information). However, the surface potential of Cr^6+^ ISM followed a decreasing trend after the reaction with the increasing concentrations of Cr^6+^ ions. The initial surface potential of the Cr^6+^ ISM was −328 mV, but after reacting with 10^−5^ m of Cr^6+^ ions, it changed to −458 mV (Figure 2i). Figure S4b (Supporting Information) shows the Gaussian distributions corresponding to the surface potential of Cr^6+^ ISM and when it is reacted with different concentrations of Cr^6+^ ions. The surface potential of As^3+^ ISM following a trend similar to Pb^2+^ ISM, exhibited an initial surface potential of −451 mV, but after reacting with 10^−5^ m of As^3+^ ions, it was increased to −315 mV (Figure S5a, Supporting Information). The corresponding Gaussian distribution is shown in Figure S5b (Supporting Information).

### 2.3 Sensing Performance, Stability, Selectivity, and Reusability Analysis of Different SL‐TENS

**Figure 3:**
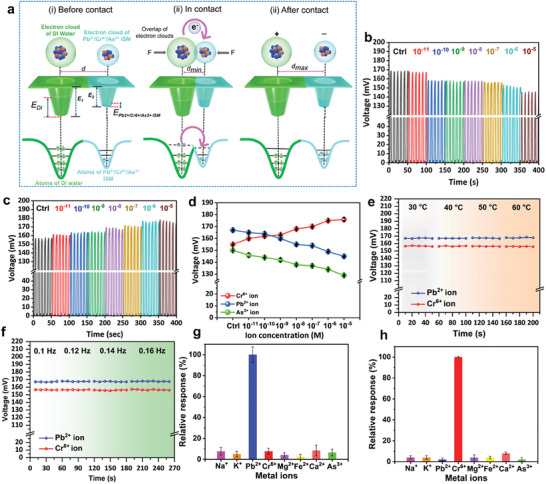
Working mechanism and sensing performance of the SL‐TENS. a) Schematic illustrations of the working principle and electron transfer process during contact electrification between ISMs and DI water in the single‐electrode configuration SL‐TENS. b,c) Output voltage signals recorded from the triboelectrification of the Pb^2+^ and Cr^6+^ ISMs with DI water before and after their reaction with the various concentrations of the Pb^2+^ and Cr^6+^ ions (10^−11^, 10^−10^, 10^−9^, 10^−8^, 10^−7^, 10^−6^, and 10^−5^ m). d) Overall output voltage trend with increasing concentration of Pb^2+^, Cr^6+^, As^3+^ions (10^−11^–10^−5^ m). e) The voltage output signal obtained for Pb^2+^ and Cr^6+^ ISMs (reacted with 10^−5^ M concentration of Pb^2+^ and Cr^6+^ ions) at increasing temperature from 30 to 60 °C. f) The voltage output signal obtained for Pb^2+^ and Cr^6+^ ISMs at increasing frequency from 0.1 to 0.16 Hz. g,h) The selectivity of the Pb^2+^ and Cr^6+^ ISMs‐based SL‐TENS for the Pb^2+^ and Cr^6+^ ion in the presence of other metal ions (Na^+^, K^+^, Mg^2+^, Ca^2+^, and Fe^2+^).

A sensing model was developed considering the binding affinity of Pb^2+^, Cr^6+^, and As^3+^ ISMs with their respective heavy metal ions. The operation of the SL‐TENS needs contact between the two layers (solid and liquid layer).^[^ ^52^, ^53^ ^]^ The ISMs reacted with the different concentrations of respective heavy metal ions and were used as the solid triboelectric layer, and DI water was used as contact liquid for solid–liquid contact electrification. DI water is a widely used contact liquid as it is a cheap, non‐toxic, and readily available solvent.^[^ ^34^, ^54^ ^]^ **Figure** **3**a demonstrates an electron cloud/potential well model to explain the contact electrification between ISMs and contact liquid DI water. The contact and separation between ISMs and DI water periodically induces the triboelectric effect, which creates a potential difference and electrostatic induction on the Cu wire electrode, resulting in a range of output voltage signals.

Before the integration of as‐fabricated SL‐TENS with the robotic hand's fingers, their sensing performance was tested by periodic contact separation of the surface of ISMs with DI water using a vertical dip coating system. Figure S6 (Supporting Information) shows the images of the contact separation process of the wire sensor with DI water. Output voltage signals were recorded from this process before and after reaction of Pb^2+^, Cr^6+^, and As^3+^ ISMs with various concentrations of the respective Pb^2+^, Cr^6+^, As^3+^ions (10^−11^, 10^−10^, 10^−9^, 10^−8^, 10^−7^, 10^−6^, and 10^−5^ m), as shown in Figures 3b,c and Figure S7a (Supporting Information). The overall output voltage trend is presented in Figure 3d. When the concentration of Pb^2+^ ion increased from 10^−11^ to 10^−5^ m, the output voltage reduced from 167 to 145 mV. Similarly, as the concentration of As^3+^ ion increased from 10^−11^ to 10^−5^ m, the output voltage dropped from 150 to 129 mV. In contrast, an increase in Cr^6+^ ion concentration (10^−11^–10^−5^ m) resulted in the output voltage rising from 155 to 176 mV. The trend in output voltage can be explained by interfacial charge transfer phenomena at the solid–liquid surface. As shown in KPFM data of Figures 2h,i and Figure S5a (Supporting Information), with the increasing concentration of Pb^2+^, Cr^6+^, and As^3+^ions, the surface potential of Pb^2+^ and As^3+^ ISMs is consistently increased, while the surface potential of Cr^6+^ ISM consistently decreased. As a result, the Pb^2+^ and As^3+^ ISMs, after reacting with their respective Pb^2^⁺ and As^3^⁺ ions, are shifted at the positive side of the triboelectric series compared to the unreacted Pb^2+^ and As^3+^ ISMs. Whereas Cr^6+^ ISM reacted with the Cr^6+^ ion and is positioned in the less positive position with respect to the unreacted Cr^6+^ ISM. The DI water is placed on the highly positive side of the triboelectric series.^[^ ^55^ ^]^ Hence, the reaction with Pb^2+^ and As^3+^ions decreases the potential difference between the respective solid triboelectric layer (Pb^2+^ and As^3+^ ISMs) and contact liquid, which ultimately reduces the interfacial charge transfer and output voltage. The reaction with the Cr^6+^ ion increases the potential difference between the Cr^6+^ ISM solid triboelectric layer and contact liquid, which ultimately increases the interfacial charge transfer and output voltage. The change in the surface potential of Pb^2+^, Cr^6+^, and As^3+^ ISMs after reaction with their respective ions indicates a change in the work function of the solid triboelectric layers. As calculated from (Figure 2e,f; Figure S3a, Supporting Information), the work functions of Pb^2+^, Cr^6+^, and As^3+^ ISMs are 5.18, 5.71, and 5.28 eV. After reaction with the respective ions, the work functions of Pb^2+^, Cr^6+^, and As^3+^ ISMs were changed to 5.25, 5.59, and 5.91 eV, respectively. The increment in the work function of Pb^2+^ and As^3+^ ISMs reduced the interfacial charge transfer which resulted in the decreased output voltage. The reduction in the work function of Cr^6+^ ISM facilitates enhanced interfacial charge transfer by overcoming the surface potential barrier leading to the increase in output voltage.

The sensing performance of the as‐developed Pb^2+^, Cr^6+^, and As^3+^ ISMs‐based SL‐TENS was also investigated by measuring the output voltage at different temperatures of the contact liquid DI water. As shown in Figure 3e, with increasing temperature from 30 to 60 °C, the voltage output signal shows no significant variation. The stable voltage output of the As^3+^ ISM‐based TENS with increasing temperature from 30 to 60 °C is shown in Figure S7b (Supporting Information). The voltage output of the different SL‐TENS was further evaluated as a function of the frequency of contact separation.^[^ ^34^ ^]^ Figure 3f and Figure S7c (Supporting Information) clearly show that the output voltage of the as‐fabricated triboelectric nanosensors remains consistent, unaffected by changes in frequency from 0.1 to 0.16 Hz, demonstrating a frequency‐independent output response. It clearly shows that rapid changes in triboelectric charges at the interface of the solid‐liquid layer have minimal impact on the performance of different SL‐TENS, even at higher values of contact frequency. Additionally, the selectivity of the as‐designed different sensors was tested in the presence of other metal ions (Na^+^, K^+^, Mg^2+^, Ca^2+^, and Fe^2+^) to validate their utilization in the natural environment. Because of the high selectivity of the ISMs toward their respective ions, other heavy metal ions do not selectively bind with the sensors, resulting in a negligible relative response voltage compared to the Pb^2+^, Cr^6+^, and As^3+^ (145, 178, 130 mV) ions when detected by their respective sensors (Figures 3g,h; Figure S7d, Supporting Information). The long‐term output stability of the solid‐liquid triboelectric nanosensors was confirmed to validate their application in real environments. The contact separation of the Pb^2+^, Cr^6+^, and As^3+^ ISMs with DI water was performed for a prolonged duration as shown in Figure S8 (Supporting Information). The results demonstrated that as‐designed sensors are highly stable, maintaining their electrical output even after prolonged operation which ensures durability. Notably, the output performance of the different SL‐TENS remains unaffected by environmental humidity variations ranging from 25 to 85%, confirming the reliability of the designed sensing platform as shown in Figure S9 (Supporting Information).

To assess the repeatability of the Pb^2+^, Cr^6+^, and As^3+^ ISMs based different SL‐TENS sensors, voltage output data was collected over a span of 10 days. The results demonstrated consistent voltage outputs throughout the 10‐day testing period, indicating that the sensors maintained their stability and functionality over time (Figure S10a, Supporting Information). Additionally, the sensors were washed using EDTA after each use to regenerate ion‐free sensors considering their real‐environmental use and tested for their sensing performance across 1 to 10 washing cycles. The repeated washing revealed minimal degradation in performance even after 10 washing cycles, showcasing the reusability of the sensors for the long term (Figure S10b, Supporting Information). These findings validate the repeatability and reusability of the sensors, ensuring their applicability in real‐environmental monitoring applications.

### 2.4 Integration of Different SL‐TENS with the Robotic Hand for On‐site Detection of Pb^2+^, Cr^6+^, and As^3+^ Ions

**Figure 4:**
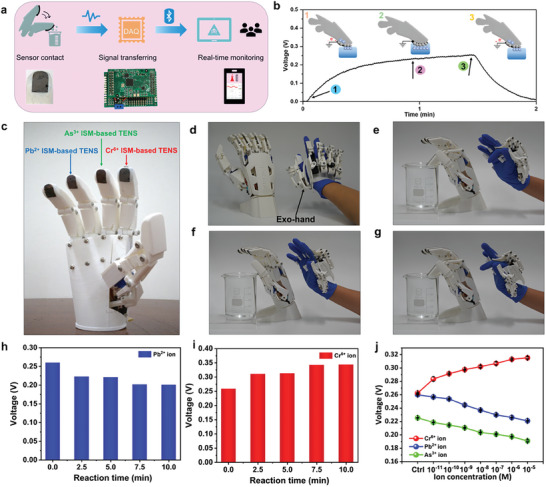
The SL‐TENS integration with the robotic hand fingers for on‐site detection. a) Schematic showing the ISM‐based SL‐TENS affixed onto the robotic hand fingers and wireless data transmission by a Bluetooth system to the smart tablet of the user in real‐time. b) The diagram outlining the voltage signal generation process during the detection of heavy metal ions by the ISM‐based robotic hand SL‐TENS. c) The photograph of the robotic hand integrated with the multiple SL‐TENS for multiplex detection of heavy metal ions. d–g) Photographs showing remote control of the contact and separation of robotic hand fingers with DI water by an exo‐hand through wireless communication technology. h,i) Reaction time test of Pb^2+^ and Cr^6+^ISMs based SL‐TENS to choose the shortest reaction time for rapid on‐site detection. j) Output voltage trend of the Pb^2+^, Cr^6+^, and As^3+^ ISMs‐based robotic hand SL‐TENS before and after reaction with different concentrations of heavy metal ions for 5 mins, when contacted and separated with DI water.

In order to integrate with the robotic fingers, the sensors were designed into a planar shape that allows better contact and more effective fitting to the fingers. The as‐designed different SL‐TENS were integrated with the robotic hand fingers to demonstrate the feasibility of their sensing performance for on‐site detection. Three sensors were affixed to the tip of the ring, index, and middle fingers of the robotic hand to detect Pb^2+^, Cr^6+^, and As^3+^ions simultaneously. Each sensor was connected to the pin of a printed circuit board (PCB), which performs signal conditioning, processing, and wireless transmission. The channels on the PCB board facilitate the detection of various analytes together without causing any interference. The Bluetooth wireless transmission system of the PCB sent the data wirelessly to the smart laptop or tablet of the user in real‐time to achieve remote monitoring of the analytes as shown in **Figure** **4**a. The details of the circuit and workflow involved in the wireless data transmission process are shown in Figure S11 (Supporting Information). Further, in Figure 4b, the diagram outlines the signal generation process during the detection of ions. Due to the different propensities of materials to gain and lose electrons, when the sensor contacts the liquid, electrons flow from the external circuit to the electrodes to balance the surface charge. This creates a potential difference between the SL‐TENS and the ground. Once the solid and liquid are no longer in motion, the number of electrons reaches equilibrium, and no electrons flow in the external circuit. When the sensor separates from the liquid, electrons flow in the opposite direction to balance the surface charge, producing a reversed electrical output. Figure 4c shows the photograph of the robotic hand integrated with the different SL‐TENS for multiplex detection of heavy metal ions. To avoid exposure to hazardous heavy metal ions during the sampling and detection process, the robotic hand was remotely controlled using an exo‐hand by wireless communication technology. As demonstrated in Figures 4d–g and Movie S1 (Supporting Information) the exo‐hand remotely controls the contact and separation of robotic finger‐based sensors with the contact liquid DI water. Wireless communication was established by utilizing the Wi‐Fi function of two Arduino development boards. Firstly, the addresses of both Arduinos are set to facilitate communication channel establishment followed by the signal transmission to the Arduino board of the robot hand through programming. Upon receiving the signal, the control board converts it into an analog signal, controlling the finger movements of the robot hand, thereby achieving remote wireless control. Figures S12,S13 (Supporting Information) shows the circuits and algorithms involved in controlling the movement of robotic hand fingers and wireless control of the movement of robotic hand fingers by the exo‐hand. Before demonstrating the sensing performance of the robotic hand‐integrated Pb^2+^, Cr^6+^, and As^3+^ ISMs‐based SL‐TENS, a reaction time test was performed (Figures 4h **,** **i**; Figure S14, Supporting Information). According to the test results, the voltage output of the developed sensors changes significantly after five, seven and a half minutes of reaction time, respectively. After 10 min, the sensor response reaches saturation and no further changes are observed. Considering the need for rapid on‐site detection in real applications, the least time duration of 5 min was chosen as the reaction time for these robotic sensors to react to the respective ion solutions. Further, Figure 4j shows the output voltage trend of these robotic hand‐based SL‐TENS before and after reaction with different concentrations of the respective heavy metal ion solutions, when contacted and separated with DI water periodically. The results show that the robotic hand‐based sensors have a similar output trend as the wire sensor, although the values differ due to the use of different shapes and sizes of the sensors and measurement system. To demonstrate the real‐time detection capability of our sensing system, a Cr^6+^ ISM‐based sensor was used for the detection of Cr^6+^ ions. As shown in Movie S2 (Supporting Information), when the concentration of the Cr^6+^ ion solution changes from 0 to 10^−5^ m, the voltage output changes from ≈250 to 310 mV.

### 2.5 Energy Harvesting by TEG to Develop Self‐powered Robotic Platform

**Figure 5:**
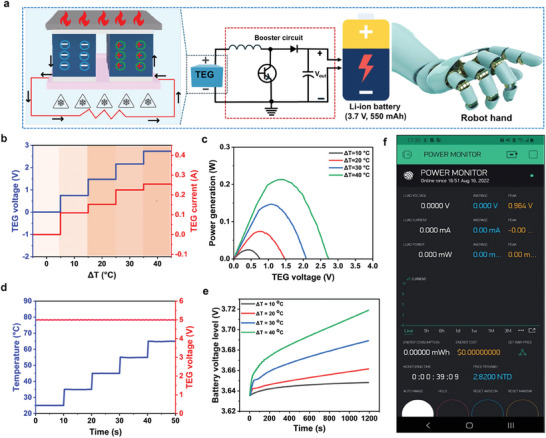
Measurement of output performance of the thermoelectric generator. a) Schematic and circuit diagram showing the TEG powering a 3.7 V, 550 mAh lithium‐ion battery for driving the robot hand. b) Voltage output and current obtained from the TEG with increasing applied temperature difference from 10 to 40 °C. c) Power output generated under different temperature gradients obtained by multiplication of open circuit voltage and short circuit current. d) The voltage output obtained from the TEG using a booster circuit under different temperature gradients. e) The battery voltage level under different temperature gradients for 1200 s charging time. f) Mobile phone app showing the power of the battery.

A commercial TEG module was utilized for harvesting thermal energy, which converts thermal energy into electrical energy by leveraging the Seebeck effect. It operates by leveraging the thermoelectric properties of Bi_2_Te_3_ materials to produce a voltage when there is a temperature difference across the module.^[^ ^56^ ^]^ To evaluate the performance of the TEG, a thermal system was employed to generate a temperature difference (ΔT) across the device which results in the generation of electrical output. The output voltage and current were measured using a commercial electric meter. **Figure** **5**a illustrates a schematic and circuit diagram for charging a lithium‐ion battery by the TEG to power the robotic hand. As shown in Figure 5b, ΔT was increased from 10 to 40 °C by considering the real environment temperature range which resulted in the rise of open‐circuit voltage (V_oc_) and short‐circuit current (I_sc_). Figure 5c shows the power output generated under different temperature gradients obtained as a product of V_oc_ and I_sc,_ where power is directly proportional to temperature difference. As the temperature difference increased, the generated power also increased. In order to charge the lithium‐ion battery to its maximum voltage, a booster circuit was used to obtain a stable voltage (5 V), as fluctuation in temperature difference can result in unstable output voltage unsuitable for charging. Figure 5d shows that voltage output will remain constant at 5 V even with different temperature gradient values. The boosted voltage was then used to charge a 3.7 V, 550 mAh rechargeable lithium‐ion battery. It is demonstrated in Figure 5e that as the temperature gradient was increased from 10 to 40 °C, the battery voltage level was also increased for 1200 s of charging duration. The output power of the battery can be monitored at any stage during the charging process. Figure 5f shows a mobile phone app interface displaying the power of the battery.

### 2.6 Detection of Pb^2+^, Cr^6+^, and As^3+^ ions in Real Samples by Respective SL‐TENS Integrated with Robotic Hand

**Figure 6:**
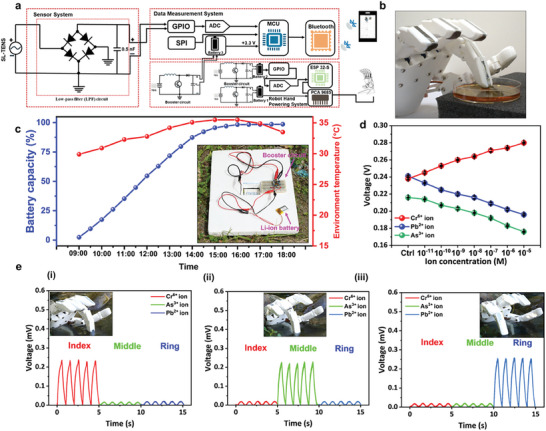
Real sample heavy metal ion detection by robotic hand‐based SL‐TENS. a) Schematic and circuit diagram showing a fully self‐powered chemical sensing system with a sensing system, data measurement system, and TEG system for powering the robot hand. b) Photograph showing the detection of heavy metal ions by robotic hand‐based sensors in lake water. c) The TEG harvests the thermal energy generated by the temperature gradient of the ground and surrounding environment for charging the lithium‐ion battery. d) Voltage output trends of Pb^2+^, Cr^6+^, and As^3+^ ISMs‐based robotic hand SL‐TENS used for detection Pb^2+^, Cr^6+^, and As^3+^ions in Drunken Moon Lake water samples spiked with their different concentrations ranging from 10^−11^ to 10^−5^ m. e (i–iii) Voltage output data obtained when Pb^2+^, Cr^6+^, and As^3+^ ISMs based SL‐TENS affixed on ring, finger, and middle fingers of the robotic hand were directly utilized for the detection heavy metal ions in lake water sample.

The Pb^2+^, Cr^6+^, and As^3+^ ISMs‐based SL‐TENS integrated with the robotic hand for detection of Pb^2+^, Cr^6+^, and As^3+^ions in lake water samples. **Figure** **6**a shows the schematic and circuit diagram of the self‐powered chemical sensing system having a sensor system, data measurement system, and TEG system for charging the battery to power the robot hand. The power consumption of the different components of the entire sensing system is provided in Table S1 (Supporting Information). Figure 6b presents the picture of the robot hand detecting heavy metal ions in a lake water sample (collected from National Taiwan University Lake). Further, the charging of the battery by the TEG was demonstrated in a real environment by keeping the system in outdoor condition. The TEG harvests the thermal energy generated by the ground temperature and ambient environmental temperature difference and fully charges the lithium‐ion battery (3.7 V, 550 mAh) in ≈9 h as shown in Figure 6c. The TEG was fixed in a thermally insulating sheet which helps to maintain a significant temperature gradient between the two surfaces by preventing the ground from heating up quickly. Figures S15a,b (Supporting Information) demonstrates variation in ground temperature and surrounding environment temperature measured at once every hour, starting from 09:00 to 18:00. Further, the sensing performance of the robotic hand‐based sensors was demonstrated for the detection of heavy metal ions in real samples. Figure 6d shows the voltage output trend when Pb^2+^, Cr^6+^, and As^3+^ ISMs‐based SL‐TENS integrated with a robotic hand detected the respective heavy metal ions spiked in different concentrations ranging from 10^−11^ to 10^−5^ m in as mentioned lake water sample. A comparison of the performance of our developed self‐powered SL‐TENS with traditional potentiometric sensors is provided in Table S2 (Supporting Information). The results indicate that our developed different SL‐TENS are highly effective at detecting Pb^2+^, Cr^6+^, and As^3+^ ions in real environmental samples, in a broad linear range (10^−11^–10^−5^ m) with detection limit (LOD) values of 5, 10, and 10 nM, respectively which is comparable to or even surpasses traditional potentiometric sensors. Subsequently, the lake water was directly used for the detection of Pb^2+^, Cr^6+^, and As^3+^ ions by robotic hand sensors. The triboelectric output voltage obtained from the detection of Pb^2+^, Cr^6+^, and As^3+^ ions was 0.253, 0.235, and 0.220 V, respectively as shown in Figure 6e. These output voltage data proved the high selectivity of the Pb^2+^, Cr^6+^, and As^3+^ ISMs‐based SL‐TENS as these sensors showed a change in output voltage only when they detected respective Pb^2+^, Cr^6+^, and As^3+^ ions. These output voltage values were out of range of the output voltage values obtained from the spike method experiment. Hence it confirmed that the Drunken Moon Lake water was not polluted by the Pb^2+^, Cr^6+^, and As^3+^ heavy metal ions. Figure S16 (Supporting Information) demonstrates the sensing performance of Pb^2+^, Cr^6+^, and As^3+^ ISMs‐based SL‐TENS integrated with a robotic hand for the detection of respective heavy metal ions such as Pb^2+^, Cr^6+^, and As^3+^ions in tap water samples spiked with their different concentrations ranging from 10^−11^ to 10^−5^ m. These results confirmed the reliability and accuracy of the robotic hand based fully self‐powered and automated chemical sensing system for on‐site and multiplexed detection of hazardous heavy metal ions in real samples. In addition, in the future, the developed SL‐TENS system can be applied for detecting heavy metal ions in various other real environment water samples. For instance, it can be used in river water to monitor industrial runoff and agricultural pollutants, as well as in ocean water to track contamination from coastal industries and shipping activities. Moreover, the system can be used in groundwater and wetland ecosystems, which will help to detect pollutants and protect sensitive environments from contamination by toxic heavy metals.

## 3 Conclusion

To summarize, this study demonstrates the development of an advanced and automated environmental monitoring platform that integrates self‐powered TENS with a robotic hand to enable automated, real‐time, and on‐site monitoring of heavy metal ions in the real environment. To overcome the issue of external power requirements, the robot hand is powered by a TEG which harvests environmental heat energy to make the chemical sensing system fully self‐powered. The TEG harvests thermal energy from ambient temperature differences and fully charges a 3.7 V, 550 mAh lithium‐ion battery in ≈9 h which is utilized to drive the robotic hand. Moreover, the robot hand was wirelessly connected to an exo‐hand for remote‐controlled sensing to avoid exposure to toxic chemicals. The TENS was composed of CuO NWs grown on copper electrodes and modified with ISM, which generates electrical output through periodic contact and separation with DI water. The high selectivity of ionophores in the ISMs allows them to bind with specific heavy metal ions, and the surface potential changes with varying ion concentrations, producing different outputs while detecting target heavy metal ions (Pb^2+^, Cr^6+^, and As^3+^). The sensing performance of the automated chemical sensing system was verified by testing real environmental samples of lake water. The as‐developed Pb^2+^, Cr^6+^, and As^3+^ ISMs‐based SL‐TENS were able to perform self‐powered detection of the respective heavy metal ions in a wide linear concentration range of 10^−11^–10^−5^ m with LOD values of 5, 10, 10 nM, respectively. The robotic hand‐based different TENS also showed excellent sensing performance in terms of long‐term operation, reusability, as well as temperature, frequency, and humidity‐independent response. Overall, the direct integration of self‐powered sensors and robotics offers automated, cost‐effective chemical sensing. This sensing system demonstrates a significant potential for water testing in real environments, protecting toxic and hazardous analytes. For example, it can be a suitable candidate for large‐scale environmental monitoring in industries such as wastewater management and drinking water systems, where continuous, automated detection of toxic ions is crucial. Furthermore, the system can be deployed in remote and hazardous environments for monitoring polluted water bodies, industrial sites, or mining areas for real‐time detection of contaminants. Its versatility and adaptability also make it a promising tool for smart agriculture and smart city applications, where it can monitor water quality and prevent contamination, thus ensuring environmental and public safety. The sensor can be modified by pathogen‐specific recognition elements, such as aptamers or antibodies for the detection of harmful pathogens. This modification would enable the monitoring of microbial contaminants in water, further expanding the application of the sensing system in environmental and public health protection.

## Conflict of Interest

The authors declare no conflict of interest.

## Author Contributions

Y.‐T.H. and A.K. contributed equally to this work. Y.‐T.H. and A.K. performed the experiments and drafted the manuscript. A.G., K.K., and S.S. performed data interpretation and revised the manuscript. Y.‐Y.C., K.‐M.L., and J.‐H.Y. provided valuable suggestions for experiment design and revised the manuscript. Z.‐H.L. conceived the original idea, supervised the project, and revised the manuscript. All authors have read and approved the article.

## Supporting information



Supporting Information

Supplemental Movie 1

Supplemental Movie 2

## Data Availability

The data that support the findings of this study are available from the corresponding author upon reasonable request.
