# Supplementary material for: Real‐Time Wireless Detection of Heavy Metal Ions Using a Self‐Powered Triboelectric Nanosensor Integrated with an Autonomous Thermoelectric Generator‐Powered Robotic System
Source: Adv Sci (Weinh). 2024 Nov 8;12(24):2410424. doi: 10.1002/advs.202410424 (PMC12199580; doi:10.1002/advs.202410424)
Supplement: Supplementary file 1 — Supporting Information [file ADVS-12-2410424-s002.docx]

**Supporting Information**

**Real-Time Wireless Detection of Heavy Metal Ions Using a Self-Powered Triboelectric Nanosensor Integrated with an Autonomous Thermoelectric Generator-Powered Robotic System**

*Yan-Tsz Huang, Arshad Khan,*

Y.-T. Huang, Y.-Y. Cheng, K.-M. Lee, J.-H. Yu

Department of Biomedical Engineering, National Taiwan University, Taipei 10167, Taiwan

Institute of Biomedical Engineering, National Tsing Hua University, Hsinchu 30013, Taiwan

A. Khan, K. Kaswan

Department of Biomedical Engineering, National Taiwan University, Taipei 10167, Taiwan

International Intercollegiate PhD Program, National Tsing Hua University, Hsinchu 30013, Taiwan

A. Ganguly

Department of Biomedical Engineering, National Taiwan University, Taipei 10167, Taiwan

S. Suresh

Department of Biomedical Engineering, National Taiwan University, Taipei 10167, Taiwan

Institute of NanoEngineering and MicroSystems, National Tsing Hua University, Hsinchu 30013, Taiwan

Z.-H. Lin

Department of Biomedical Engineering, National Taiwan University, Taipei 10167, Taiwan

Department of Power Mechanical Engineering, National Tsing Hua University, Hsinchu 30013, Taiwan

Email: [zhlin@ntu.edu.tw](mailto:zhlin@ntu.edu.tw)

**Table of Content**

1. Experimental Section
   1. Chemicals
   2. Growth of CuO NWs on Cu Wire Electrode and ISM Modification
   3. SL-TENS Sensing Process
   4. Fabrication and Integration of the Pb, Cr, and As ISMs Based SL-TENS with the Robotic Hand
   5. Energy Harvesting by Thermoelectric Generator
   6. Detection of Heavy Metal Ions by Pb^2+^, Cr^6+^, and As^3+^ ISMs based Robotic Hand SL-TENS
   7. Design and Development of a Wearable Exo-Hand for Remote Control Sensing
   8. Pb^2+^, Cr^6+^, and As^3+^ ISMs Based Robotic Hand SL-TENS for Detection of Cr^6+^, Pb^2+^ and As^3+^ Ions in Real samples
   9. Characterization of SL-TENS and Electrical Measurements

2. Supporting Tables

3. Supporting Figures

4. Supporting Movies

# **1. Experimental Section**

## **1.1 Chemicals**

Copper wire (diameter-1 mm), copper foil (thickness 127 µm), 1-Nitro-2-(n-octyloxy) Benzene, Lead (Ⅱ) Acetate Trihydrate were purchased from Alfa Aesar. Sodium Tetraphenylborate, Polyvinyl chloride, Lead Ionophore IV, Potassium Dichromate, 5,10,15,20-Tetrakis(4-methoxyphenyl)-21H,23H-Porphine Cobalt (II), Arsenic Trioxide were purchased from Sigma-Aldrich. Tetrahydrofuran (without stabilizer) and Quinaldine Red were purchased from Michigan Biotechnology Institute and Spectrum, respectively. Ultra-pure DI water was received from a Milli-Q ultrapure system, acetone and isopropanol from ECHO Chemical Co. were utilized for the experiments.

## **1.2 Growth of CuO NWs on Cu Wire Electrode and ISM Modification**

CuO NWs was grown on a Cu wire by thermal oxidation process. Before the growth process, Cu wires were cut into 3 cm length. The 1 cm part at the one end was used as electrode and the remaining 2 cm part was covered with aluminum tape. Further, the as-prepared electrodes were cleaned with DI water, ethanol, and acetone sequentially to remove surface impurities. In the next step, the 1 cm part was immersed in 1M hydrochloric acid for 10 min for removing oxides and other impurities from the surface. Lastly, Cu wires were kept for calcination in a high temperature oven at 500 $℃$ for 5 hours which resulted in the formation of a dense array of CuO NWs on the Cu wire surface.

Prior to the surface modification, ISM solutions were prepared by dissolving different components in tetrahydrofuran (THF) such as Poly vinyl chloride (PVC) as a substrate, plasticizer 2-Nitrophenyl octyl ether (2NOE) for mechanical properties of the membrane, and sodium tetraphenylborate (NaTPB) as anion to enhance ion exchange performance. Ionophores (lead ionophore IV (tert-butylcalix[4]arene-tetrakis(N,N-dimethylthioacetamide)), Quinaldine red, and 5,10,15,20-Tetrakis(4-methoxyphenyl)-21H,23H-porphine cobalt(II)) are added to these compositions which enable the ISMs to capture Pb^2+,^ Cr^6+^, and As^3+^ ions in the prepared solutions of Pb(C_2_H_3_O_2_)_2,_ K_2_Cr_2_O_7_, and As_2_O_3_, respectively. Then, Cu wire electrode surfaces containing CuO NWs were drop coated with ISM solutions and allowed to react at room temperature for 24 hours.

## **1.3 SL-TENS Sensing Process**

In order to selectively detect Pb^2+,^ Cr^6+^, and As^3+^ ions, the as-prepared ISM coated CuO NWs grown Cu wire (Pb^2+^, Cr^6+^, and As^3+^ ISMs based SL-TENS) were first reacted with different concentration of ion solutions (10^-11^ M, 10^-10^ M, 10^-9^ M, 10^-8^ M, 10^-7^ M, 10^-6^ M, and 10^-5^ M) at room temperature for 1 hour. Next, the surfaces of these sensors were cleaned with DI water to remove any unreacted heavy metal ions. For the sensing experiment, metal ions reacted SL-TENS were used as solid triboelectric layer and DI water as the contact liquid solvent. To generate the electrical output, the sensor system operated as single electrode configuration in which a vertical dip coating system (Sadhudesign, DX-5A) was utilized for periodic contact separation of solid and liquid layer. The SL-TENS sensing performance was evaluated by measuring the variation in output voltage, the signal was converted from AC to DC using a bridge rectifier and recorded by a portable Wi-Fi enabled data recorder named Elite System (BioPro Scientific Co., Ltd) for signal acquisition. Additionally, ceramic capacitors (0.5 nF) were used to obtain stable signal by removing ambient noise.

## **1.4 Fabrication and Integration of the Pb^2+^, Cr^6+^, and As^3+^ ISMs** **Based SL-TENS with the Robotic Hand**

A Cu foil was used as both substrate and electrode material for fabrication of highly flexible and ultrathin planar triboelectric nanosensors. Firstly, the different SL-TENS were fabricated by ISM coating on CuO NWs grown on a 1.85$\times$ 1.2 cm^2^ Cu foil using the previously described protocol. Following the growth process, the conducting wires were connected on the back side of the sensor using aluminum foil for the purpose of measuring electrical signals. Subsequently, a non-conductive double-sided tape was used to affix the sensors onto the robot fingers.

## **1.5 Energy Harvesting by Thermoelectric Generator**

Commercial TEG module (55x55 mm, Patel Magnets & Electronics) with 127 pairs of Bi_2_Te_3_ bulks was used for thermal energy harvesting. The experimental setup involved creating a controlled temperature gradient across the TEG module using a thermal system with hot and cold ends, while measuring electrical outputs with a commercial electric meter. Further, a booster circuit (DC-DC Boost Converter Step Up Module, DIYElectronic) was used to boost the generated electrical output. The boosted TEG voltage was then utilized to charge a lithium-ion battery (Rechargeable Li Polymer Battery, 3.7 V, 550 mAh, Brother Energy Co., Ltd.). and stored to power the robot hand. For demonstrating the application in real environment, the TEG was kept outdoors to charge the lithium-ion battery by converting ambient temperature difference into electricity for powering the robot hand.

## **1.6 Detection of Heavy Metal Ions by Pb^2+^, Cr^6+^, and As^3+^ ISMs based Robotic Hand SL-TENS**

Prior to the sensing experiment, reaction time test of the different SL-TENS was performed. These SL-TENS sensors reacted with their respective (Pb^2+^, Cr^6+^ and As^3+^) ion solutions for different time periods (0, 2.5, 5.0, 7.5, and 10 minues). Reaction time of 5 min was chosen for further sensing experiment. The sensors were integrated with index, middle, and ring fingers of the robot hand. An Arduino microcontroller (NXP Semiconductors, PCA9685-PWM) was utilized to control the movement of the robotic fingers, enabling them to undergo repetitive contact and separation cycles with DI water for generating the triboelectric effect. The contact separation time between the sensor and DI water was set up as 1s for each cycle. The output generated from the contact electrification between the sensors and DI water was sent wirelessly to the laptop using a 9 × 5.5 cm^2^ electronic PCB with a 12-bit analog-to-digital converter. The main part of the sensing system through which robotic sensing fingers send data wirelessly is the CYBLE-214009-00 BLE module (Cypress Semiconductor Corp). It uses a CC2640 controller (Texas Instruments) and has Bluetooth low energy (BLE) built-in for wireless communication.

## **1.7 Design and Development of a Wearable Exo-Hand for Remote Control Sensing**

The different parts of exo-hand were designed by 3D design software and produced using 3D printing technology followed by assembly using screws. Variable resistors, essential for precise monitoring of finger flexion and extension were strategically integrated into the manipulator's joints to accurately detect finger trajectory and posture. The wireless communication between the exo-hand and robotic hand was established by Wi-Fi function of two Arduino development boards for remote sensing. The signal from the exo-hand was transmitted to the Arduino board of the robotic hand, where it was converted into an analog signal to control robotic fingers movements.

## **1.8 Pb^2+^, Cr^6+^, and As^3+^ ISMs** **Based Robotic Hand SL-TENS for Detection of Cr^6+^, Pb^2+^ and As^3+^ Ions in Real samples**

Detection of Cr^6+^, Pb^2+^ and As^3+^ ions was performed in real sample such as lake water to demonstrate the robotic fingers integrated sensors performance for automated and on-site detection in real environment. The lake water was collected from the drunken moon lake, National Taiwan University, Taipei, Taiwan. Firstly, these samples were centrifuged and filtered to remove impurities. Next, different concentrations of Cr^6+^, Pb^2+^ and As^3+^ ions were spiked onto the lake water sample. Subsequently, the robotic sensing fingers with different S-L TENS surface were directly contacted and separated with the above sample for the detection of Cr^6+^, Pb^2+^ and As^3+^ ions by generating triboelectric effect. The triboelectric output voltage from each sample was recorded and transmitted wirelessly via the Bluetooth system to user’s laptop or tablet device.

## **1.9 Characterization of SL-TENS and Electrical Measurements**

The surface morphology of the as-grown CuO NWs and Pb^2+^, Cr^6+^, and As^3+^ ISMs were characterized by using a field emission scanning electron microscope (FESEM, JEOL JSM-7000F). A high-resolution X-ray photoelectron spectroscopy (HXPS, ULVAC-PHI, Quantes) was used to verify the binding of ISMs to respective Cr^6+^, Pb^2+^ and As^3+^ ions. The work function of as-synthesized solid triboelectric layer was measured by ultraviolet photoelectron spectroscopy (UPS) with He I as the UV source. The work function of the Pb^2+^, Cr^6+^, and As^3+^ ISMs before and after reaction with 10^-5^ M concentration of Cr^6+^, Pb^2+^ and As^3+^ ions solutions was calculated using the formula $\Phi$ = 21.22 − (*E*_Fermi_ – *E*_Cut-off_), where *E*_Cut-off_ is the secondary electron cut-off energy, *E*_Fermi_ is the Fermi energy, $\Phi$ is the work function, and 21.22 is the energy of UV source. Next, amplitude modulation Kelvin probe force (AM-KPFM, Bruker, Icon-PT) was employed to measure the surface potential of Pb^2+^, Cr^6+^, and As^3+^ ISMs after reaction with different concentration of Cr^6+^, Pb^2+^ and As^3+^ ions solutions.

# **Supporting Tables**

| Component | Voltage Requirement | Current Draw (mA) | Power Consumption (W) | Power Source | Battery Life (3.7V, 550 mAh) |
| --- | --- | --- | --- | --- | --- |
| SL-TENS Sensors | No external power | - | - | Powered by harvested energy by triboelectrification | Self-powered |
| Low-pass filter  (LPF) circuit | No external power | - | - | - | No power |
| Micro Servo 9g (SG90) | 4.8 V to 6 V | 100-250 mA | 1.25 W | 3.7V, 550 mAh Li-ion battery | More than 2 hours for one servo |
| PCA9685 16-Channel PWM Servo Driver | 2.3V - 5.5V | 1 mA | Depend upon servo | 3.7V, 550 mAh Li-ion battery | More than 2 hours for one servo |
| ESP32-S Microcontroller (Wi-Fi transmit) | 3.3 V | 160-240 mA | 0.528 W | 3.7V, 550 mAh Li-ion battery | More than 2 hours for one servo |
| ESP32-S Microcontroller  (Wi-Fi receive) | 3.3 V | 80 mA | 0.26 W | 3.7V, 550 mAh Li-ion battery | More than 2 hours for one servo |
| Bluetooth Module (Embedded in PCB) | 3.3 V | 500 mA | 1.6 W | 3.7V, 550 mAh Li-ion battery | More than 2 hours |

**Table S1.** Power consumption of the different components of the sensing system.

| Sensing platform | Limit of detection | Linear range | Response time | Repeatability | Selectivity | Ion | Automated and self-powered | On-site detection | Ref |
| --- | --- | --- | --- | --- | --- | --- | --- | --- | --- |
| Ion exchange transducer | 0.414 µM | 100 µM -10 M | 5 s | Yes | Yes | Pb^2+^ | No | No | [1] |
| Paper substrate | 100 μM | 9.55-631 µM | ~21 s | Yes | Yes | Pb^2+^ | No | Yes | [2] |
| Ion-selective electrode | 10 μM | 10- 631 µM | 60 s | Yes | Yes | Pb^2+^ | No | Yes | [3] |
| Aptasensor | 8.5 pM | 10 pM-1 μM | 100 s | Yes | Yes | Pb^2+^ | No | Yes | [4] |
| Graphite-Epoxy sensor | 0.63 μM | 1 μM-10 mM | ~18 s | Yes | Yes | Cr^6+^ | No | Yes | [5] |
| carbon screen-printed electrode | 210 nM | 316 nM- 100 µM | ~20 s | Yes | Yes | Cr^6+^ | No | Yes | [6] |
| Graphene paste electrode | 8 pM | 25-34 nM | - | Yes | Yes | As^3+^ | No | Yes | [7] |
| Membrane electrode | 0.5 μM | 1 μM-1M | 30 s | Yes | Yes | As^3+^ | No | Yes | [8] |
| Aptasensor array | 0.17 pM | 2.5 pM to 2.5 μM | 200 s | Yes | Yes | As^3+^ | No | Yes | [9] |
| Ion selective membrane | 5 nM | 10 pM-10 μM | 550 ms | Yes | Yes | Pb^2+^ | Yes | Yes | This work |
|  | 10 nM |  |  |  |  | Cr^6+^ |  |  |  |
|  | 10 nM |  |  |  |  | As^3+^ |  |  |  |

**Table S2.** A summary of the traditional potentiometric sensors in comparison with our SL-TENS.

# **3. Supporting Figures**


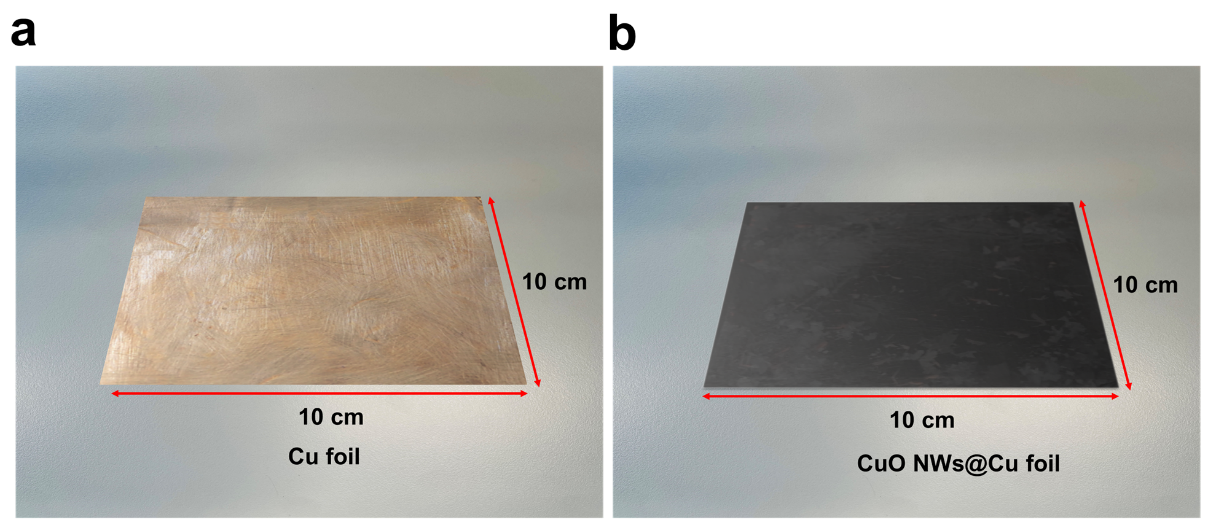


**Figure S1.** Large scale synthesis of CuO NWs on Cu foil (a) a 10 cm × 10 cm Cu foil (b) Growth of CuO NWs on Cu foil by thermal oxidation process.


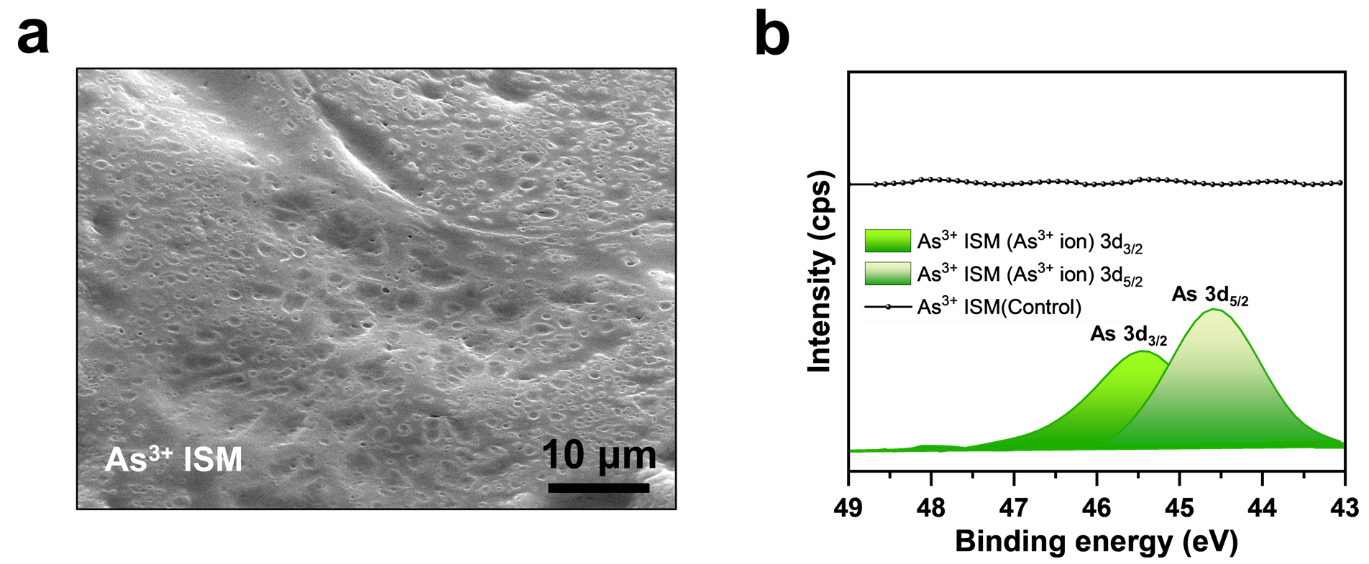


**Figure S2.** (a) Surface morphology of As^3+^ ISM showing uniform film with porous structures revealed by FESEM. (b) High resolution XPS showing As 3d peaks at 45.8 and 44.7 eV binding energy positions after reaction of As^3+^ ISM with As^3+^ ion.


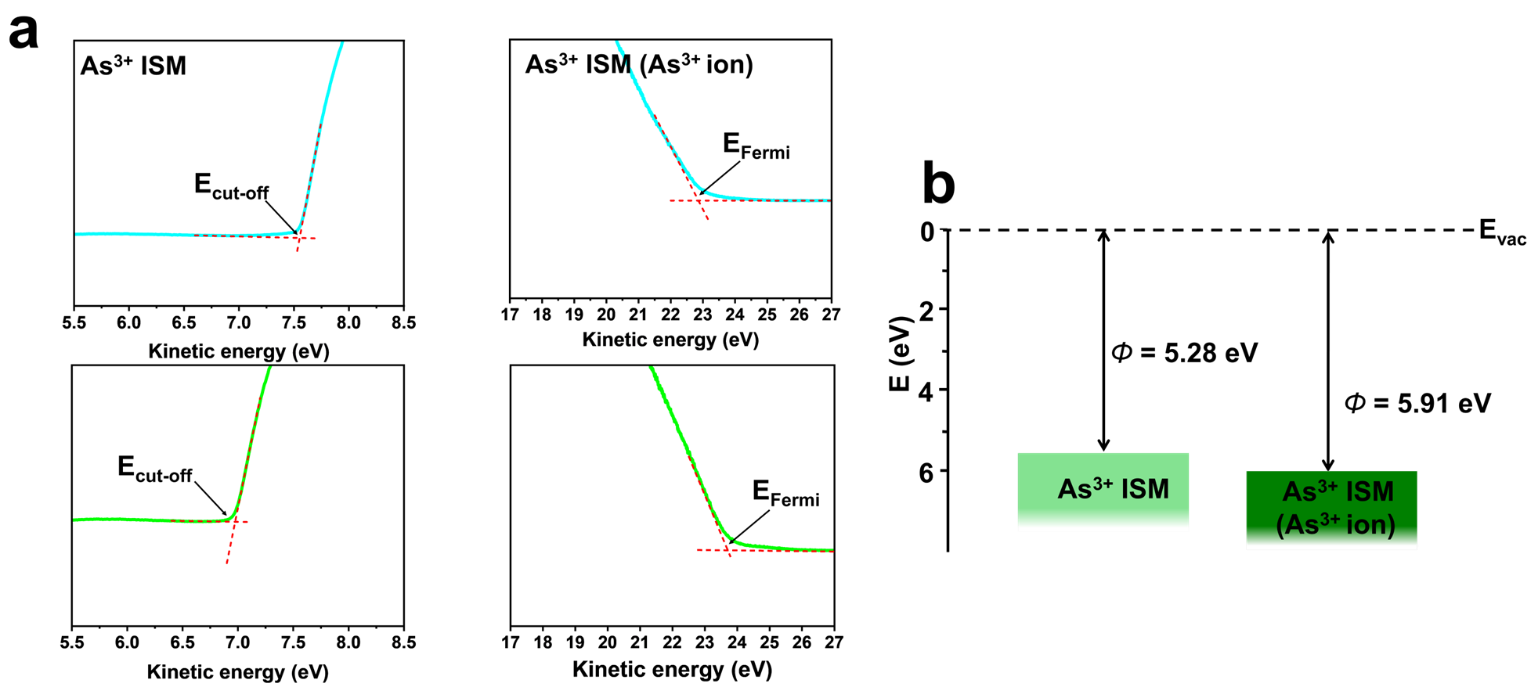


**Figure S3.** (a) The UPS spectra of As^3+^ ISM after its binding with As^3+^ ion. The positions of the indicated Fermi energy (*E*_Fermi_) and the secondary electron cutoff (*E*_Cut-off_) are utilized to calculate the work functions (Φ) of the materials. (b) A graphical illustration showing the change in the work function of As^3+^ ISM after binding with As^3+^ ion.


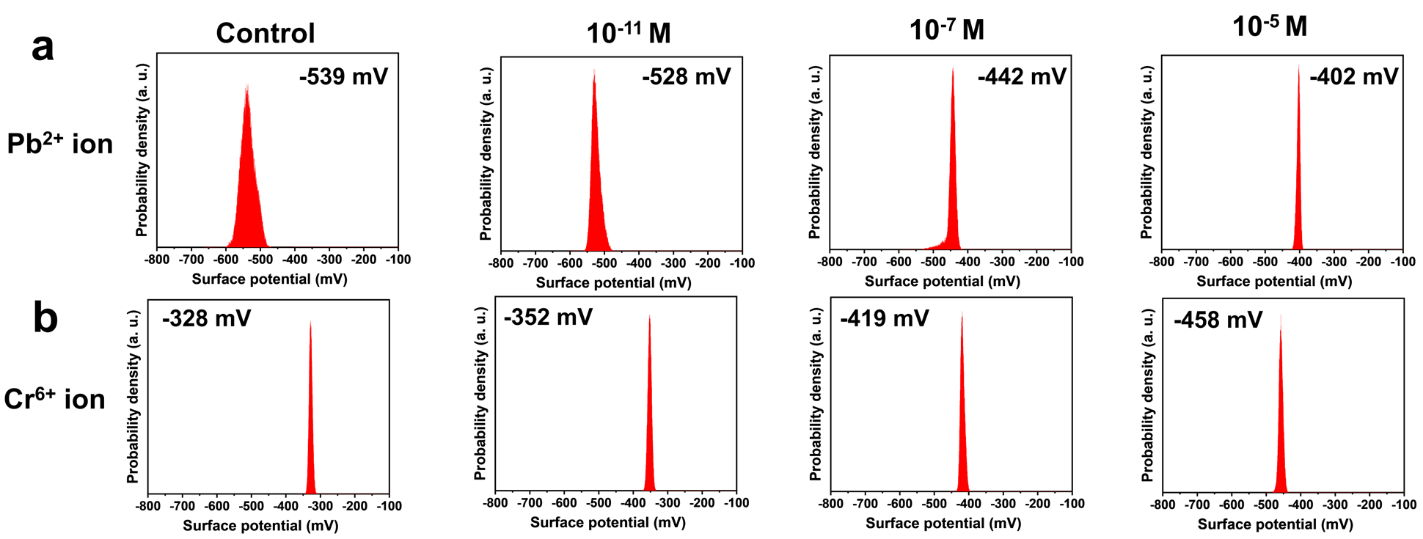


**Figure S4.** (a,b) Gaussian distribution of surface potentials after binding of Pb^2+^ and Cr^6+^ ISMs with different concentrations of Pb^2+^ and Cr^6+^ ions.


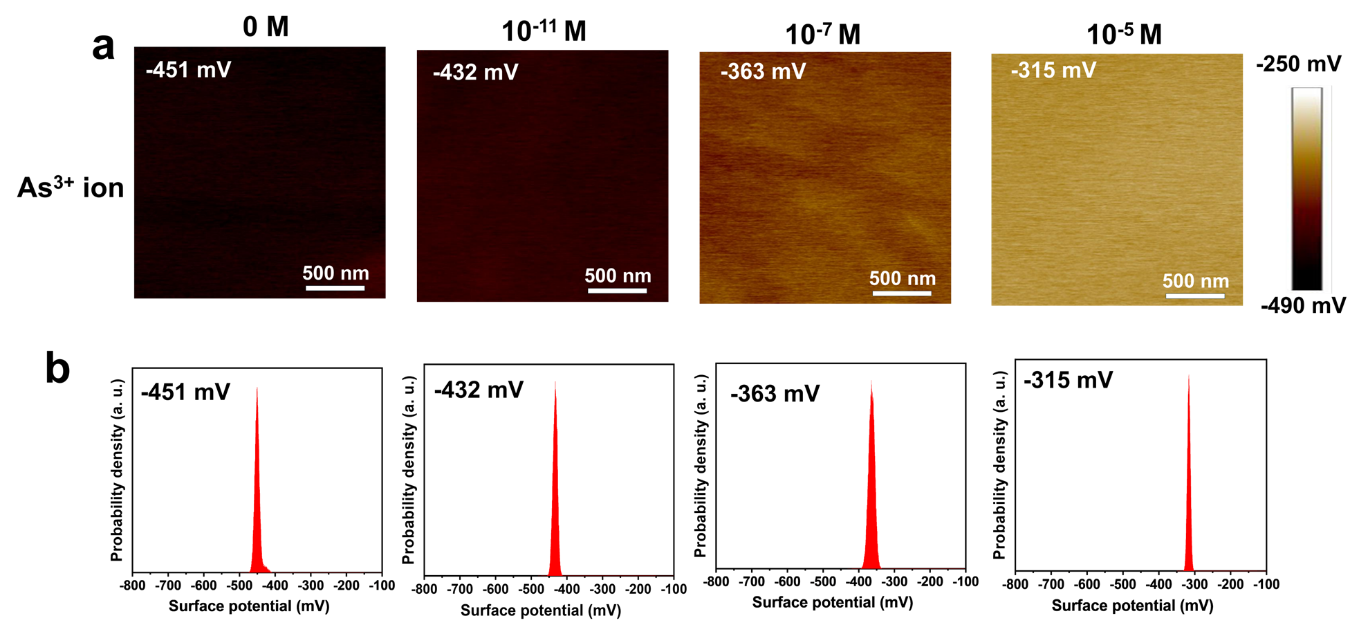


**Figure S5.** Surface potentials and corresponding Gaussian distributions of As^3+^ ISM measured by KPFM after its binding with different concentration of As^3+^ ion (0M, 10^-11^ M, 10^-7^ M, 10^-5^ M).


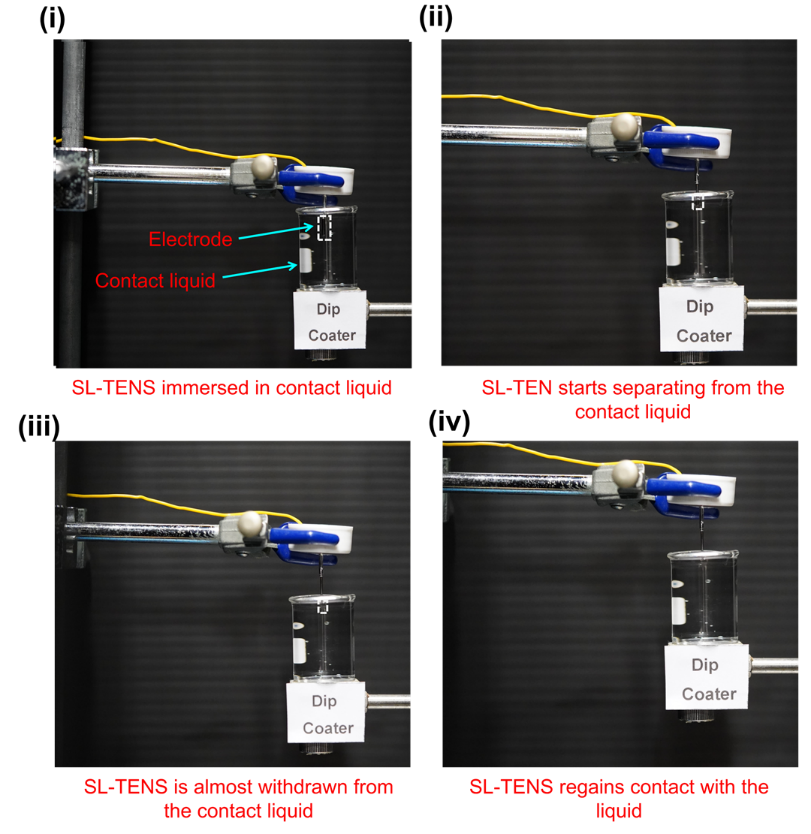


**Figure S6.** The contact separation process of the wire shaped TENS with contact solvent DI water.


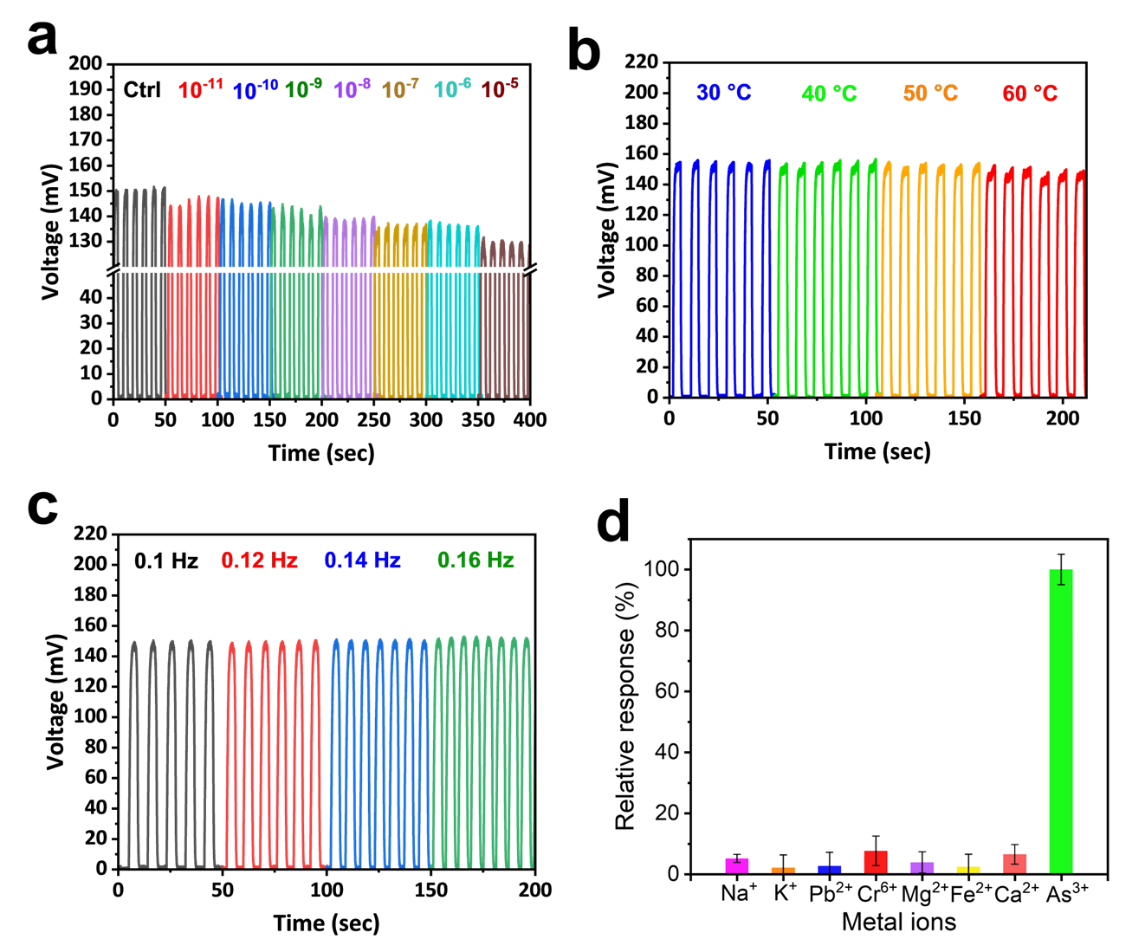


**Figure S7.** (a) Output voltage signals recorded from the contact and separation of the As^3+^ ISM with DI water before and after its reaction with the various concentrations of As^3+^ ions (10^-11^ M, 10^-10^ M, 10^-9^ M, 10^-8^ M, 10^-7^ M, 10^-6^ M, and 10^-5^ M). (b) The voltage output signal obtained for As^3+^ ISM at increasing temperature from 30 °C to 60 °C. (d) The voltage output signal obtained for As^3+^ ISM at increasing frequency from 0.1 Hz to 0.16 Hz. (e) The selectivity of the As^3+^ ISM based SL-TENS for the As^3+^ ion in the presence of other interfering metal ions (Na^+^, K^+^, Mg^2+^, Ca^2+^, and Fe^2+^).


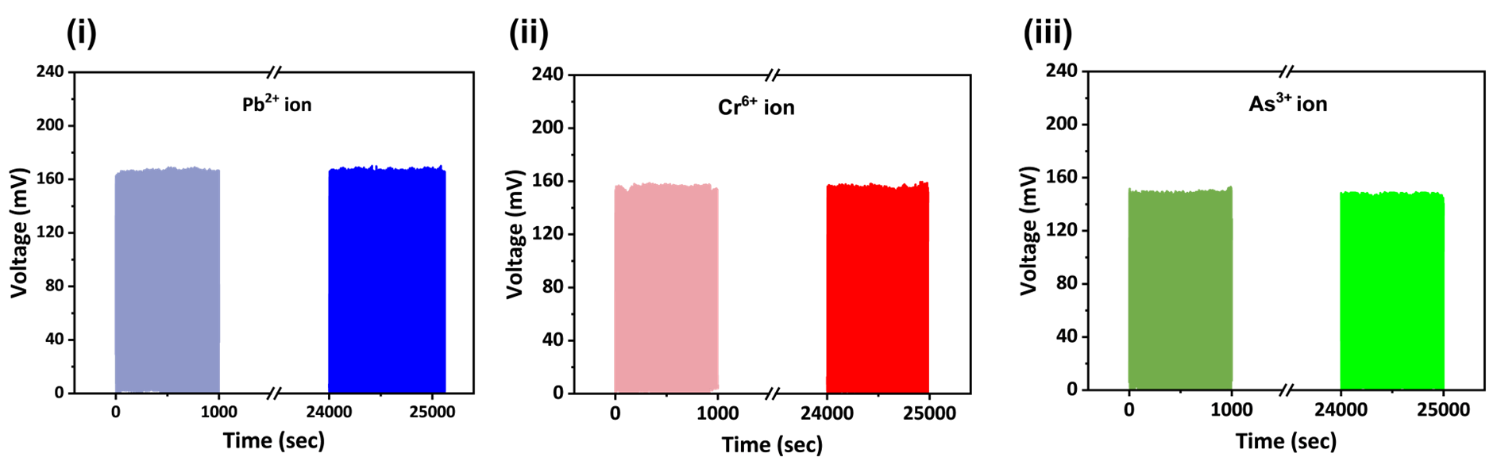


**Figure S8.** Long-term operation output stability of the Pb^2+^, Cr^6+^, and As^3+^ ISMs based different SL-TENS.


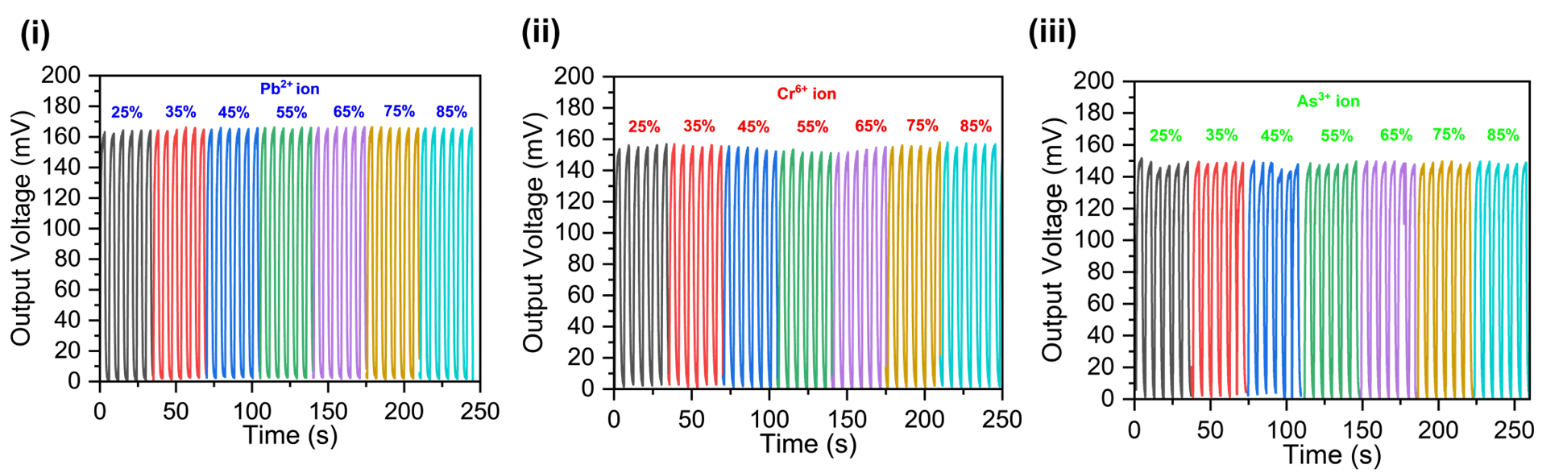


**Figure S9.** Output stability of the Pb^2+^, Cr^6+^, and As^3+^ ISMs based different SL-TENS under varying percentages of relative humidity.


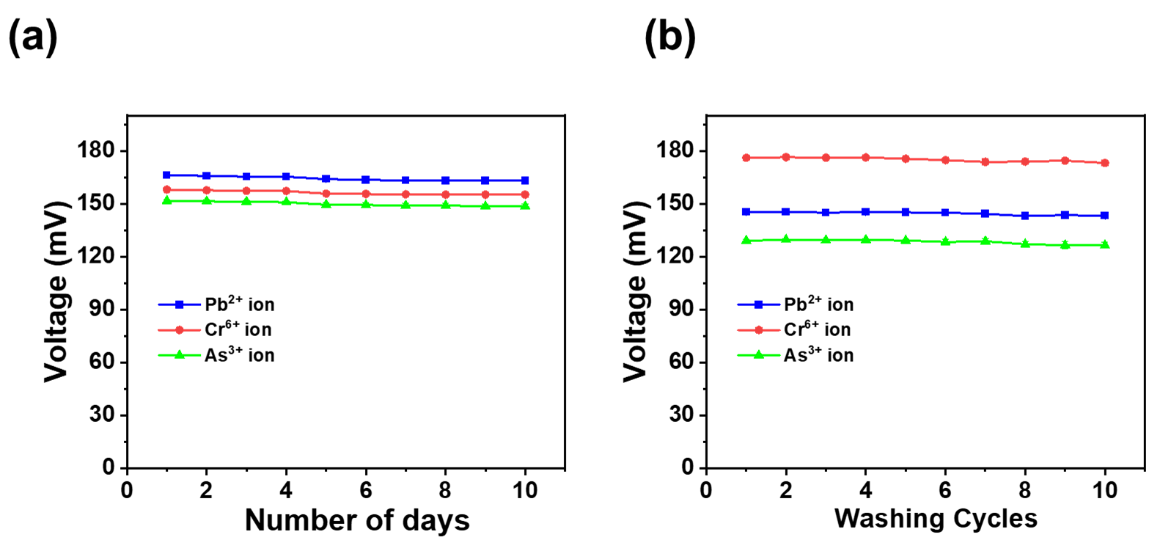


**Figure S10.** (a) Repeatability test of the Pb^2+^, Cr^6+^, and As^3+^ ISMs based different SL-TENS. (b) Reusability study of the Pb^2+^, Cr^6+^, and As^3+^ ISMs based different SL-TENS for 10^-5^ M concentration of their respective ions.


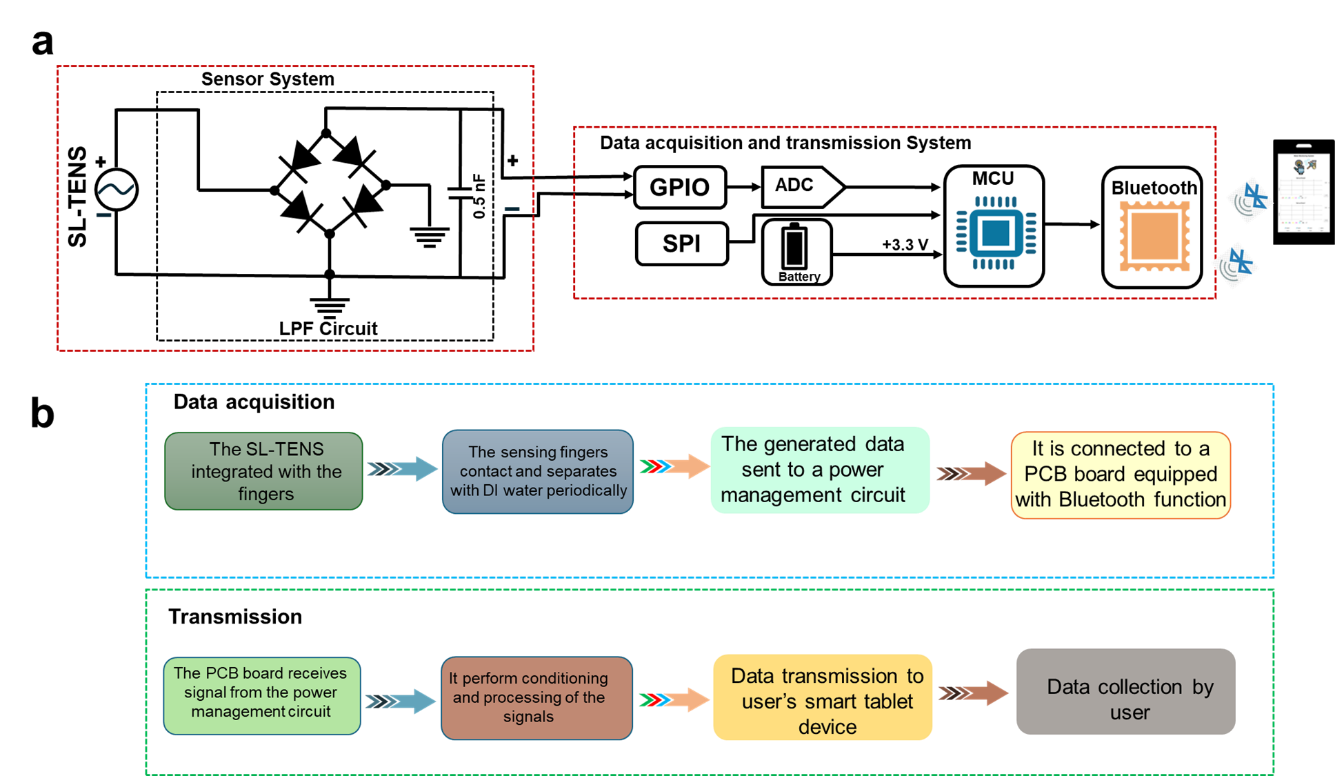


**Figure S11.** (a, b) circuit and workflow of the wireless data transmission process.


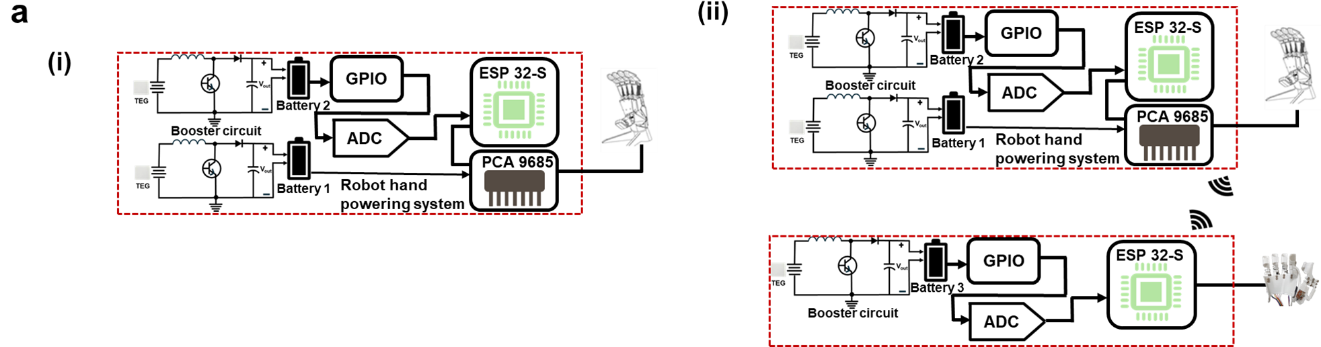


**Figure S12.** a(i, ii) Circuits for controlling the movement of robotic hand fingers and wireless control of the movement of robotic hand fingers by the exo-hand.

**
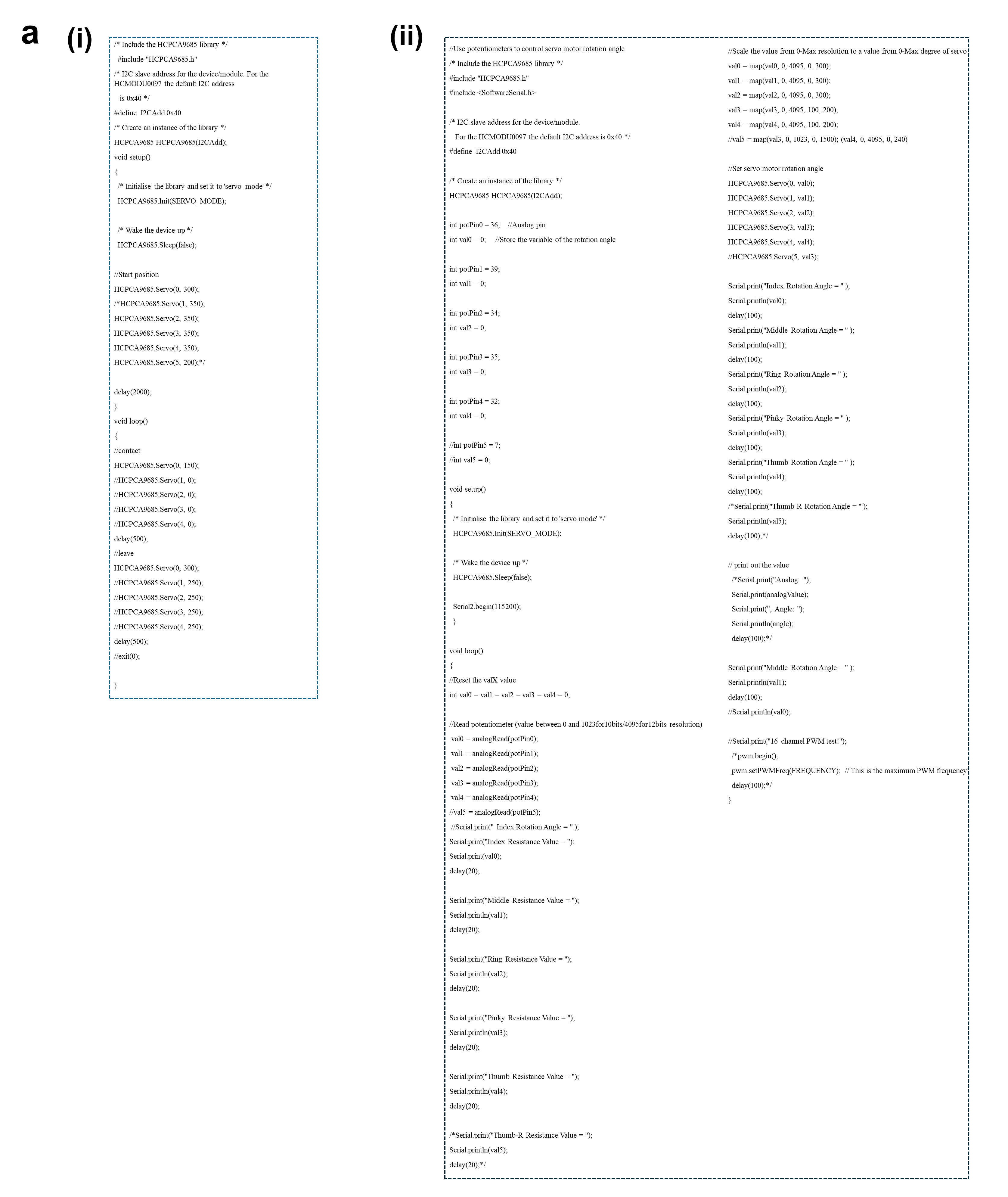
**

**Figure S13.** a(i, ii) Algorithms for controlling the movement of robotic hand fingers and wireless control of the movement of robotic hand fingers by the exo-hand.


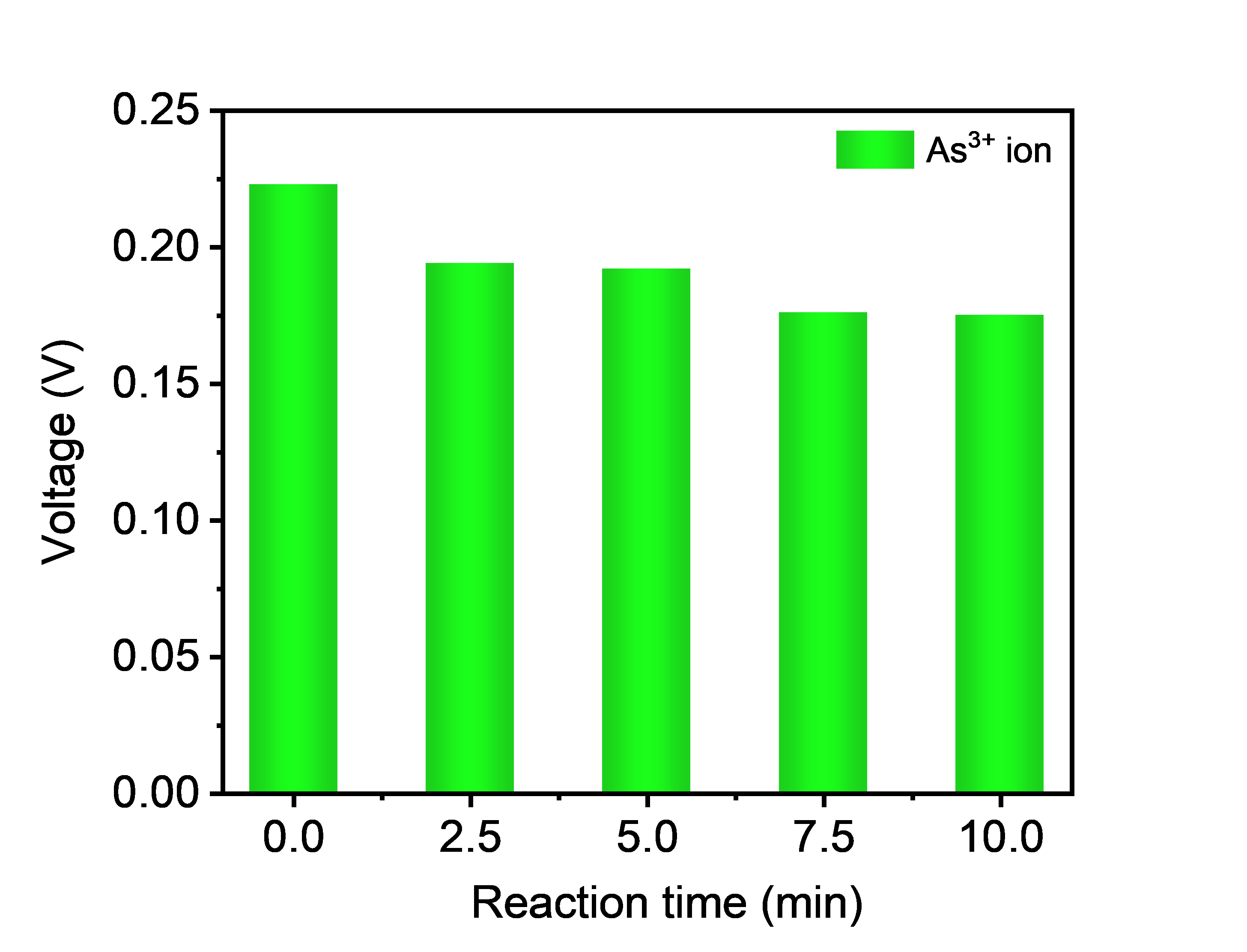


**Figure S14.** Reaction time test of AS^3+^ ISM-based Robotic hand SL-TENS to choose shortest reaction time for the purpose of rapid on-site detection.


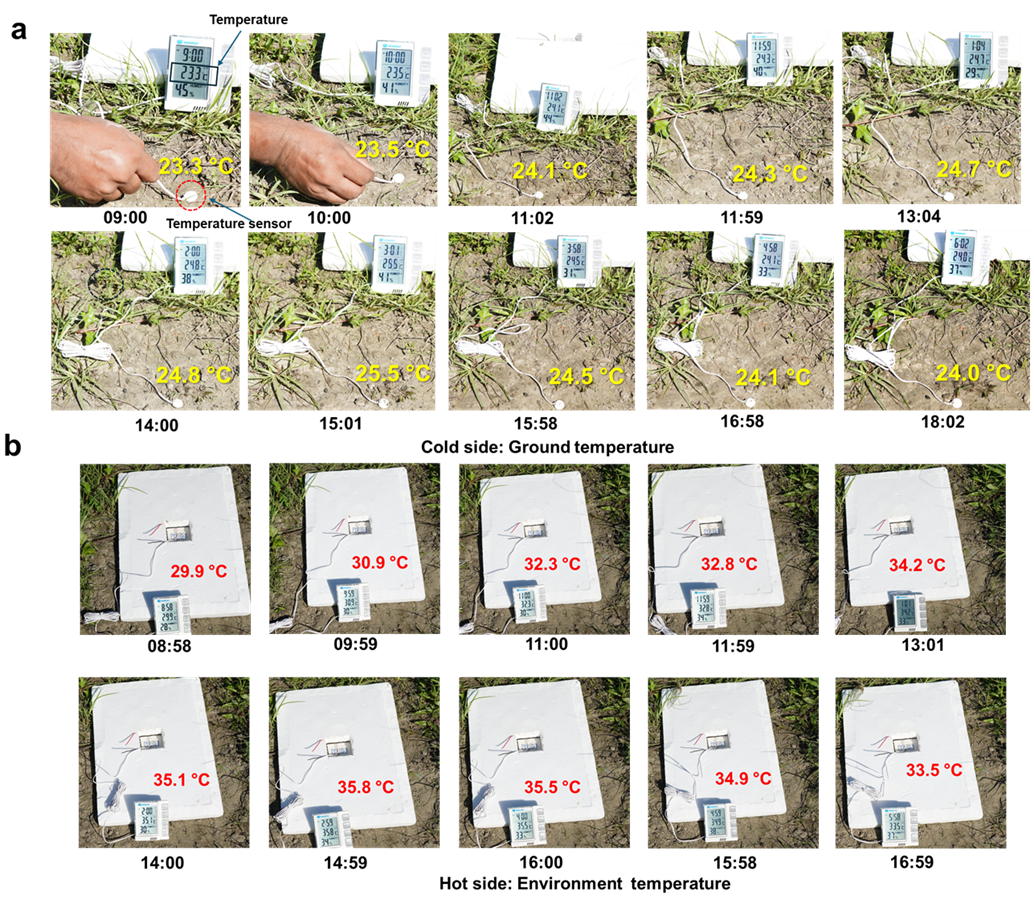


**Figure S15.** (a, b) variation in ground temperature and surrounding environment temperature measured once in every hour, starting from 09:00 to 18:00.


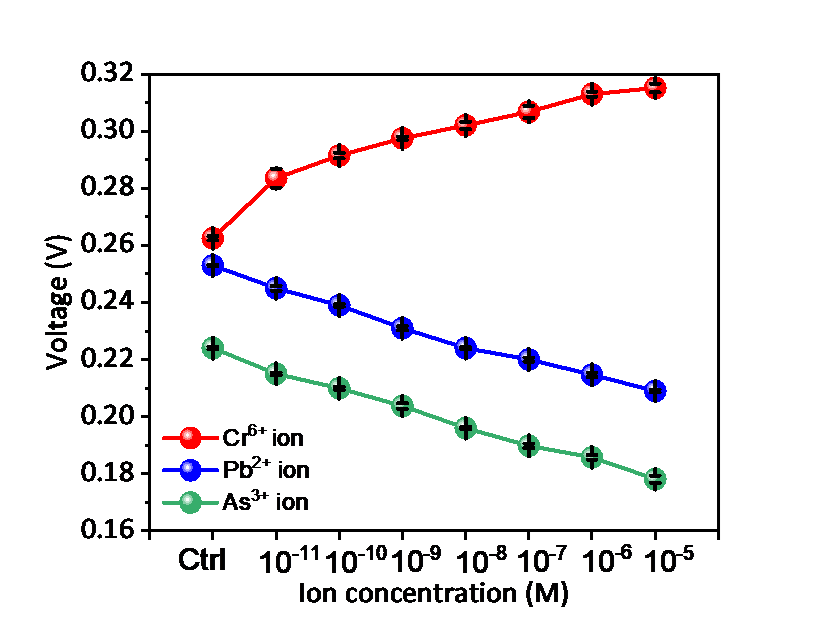


**Figure S16.** Output voltage trend of Pb^2+^, Cr^6+^, and As^3+^ ISMs based Robotic hand TENS used for detection of heavy metal ions in tap water samples spiked with their different concentrations ranging from 10^-11^ M to 10^-5^ M.

# **4.** **Supporting Movies**

**Movie S1.** Demonstration of remote control of the contact and separation of robotic hand fingers with DI water by an exo-hand though wireless communication technology.

**Movie S2.** The real-time detection of the Cr^6+^ ion by Cr^6+^ ISM based SL-TENS integrated with the robotic hand.

**References**

[1] B. E. Keshta, H. Yu, L. Wang, M. A. Uddin, H. G. El-Attar, A. E. Keshta, A. H. Gemeay, F. Hassan, S. M. Eid, *Chemical Engineering Journal* **2024**, *485*, 150049.

[2] R. Silva, A. Ahamed, Y. H. Cheong, K. Zhao, R. Ding, G. Lisak, *Analytica Chimica Acta* **2022**, *1197*, 339495.

[3] R. Ding, V. Krikstolaityte, G. Lisak, *Sensors and Actuators B: Chemical* **2019**, *290*, 347.

[4] W. Tang, J. Yu, Z. Wang, I. Jeerapan, L. Yin, F. Zhang, P. He, *Analytica Chimica Acta* **2019**, *1078*, 53.

[5] R. A. Sánchez-Moreno, M. J. Gismera, M. T. Sevilla, J. R. Procopio, *Sensors and Actuators B: Chemical* **2010**, *143*, 716.

[6] R. A. Sánchez-Moreno, M. J. Gismera, M. T. Sevilla, J. R. Procopio, *Analytical and Bioanalytical Chemistry* **2010**, *397*, 331.

[7] B. J. Sanghavi, N. S. Gadhari, P. K. Kalambate, S. P. Karna, A. K. Srivastava, *Microchimica Acta* **2015**, *182*, 1473.

[8] T. Alizadeh, M. Rashedi, *Analytica Chimica Acta* **2014**, *843*, 7.

[9] W. Tang, Z. Wang, J. Yu, F. Zhang, P. He, *Analytical Chemistry* **2018**, *90*, 8337.
